# Two-color nanoscopy of organelles for extended times with HIDE probes

**DOI:** 10.1038/s41467-020-17859-1

**Published:** 2020-08-26

**Authors:** Ling Chu, Jonathan Tyson, Juliana E. Shaw, Felix Rivera-Molina, Anthony J. Koleske, Alanna Schepartz, Derek K. Toomre

**Affiliations:** 1 Department of Cell Biology, Yale University School of Medicine, New Haven, CT USA; 2 Department of Chemistry, Yale University, New Haven, CT USA; 3 Department of Molecular Biophysics and Biochemistry, Yale University, New Haven, CT USA; 4 Department of Neuroscience, Yale School of Medicine, New Haven, CT USA; 5 Department of Molecular, Cellular, and Developmental Biology, Yale University, New Haven, CT USA; 6 Present Address: Department of Chemistry, University of California, Berkeley, CA USA

**Keywords:** Super-resolution microscopy, Fluorescent dyes, Cellular imaging, Chemical biology

## Abstract

Performing multi-color nanoscopy for extended times is challenging due to the rapid photobleaching rate of most fluorophores. Here we describe a new fluorophore (Yale-595) and a bio-orthogonal labeling strategy that enables two-color super-resolution (STED) and 3D confocal imaging of two organelles simultaneously for extended times using high-density environmentally sensitive (HIDE) probes. Because HIDE probes are small, cell-permeant molecules, they can visualize dual organelle dynamics in hard-to-transfect cell lines by super-resolution for over an order of magnitude longer than with tagged proteins. The extended time domain possible using these tools reveals dynamic nanoscale targeting between different organelles.

## Abstract (web summary)

High density environmentally sensitive (HIDE) probes allow for long time-lapse super-resolution imaging of live cells. Here the authors develop a second HIDE probe with a bio-orthogonal labelling strategy to enable two-color nanoscopy of two organelles over extended periods.

## Introduction

Super-resolution microscopy (“nanoscopy”) can visualize cellular components with resolutions as high as ~10 nanometers, far below the Abbe diffraction limit^1^. When combined with multi-color labeling strategies, nanoscopy can reveal key features of organelle structure and interactions that are hidden when visualized using diffraction-limited approaches^2,3^.

However, visualizing organelle dynamics at super-resolution remains challenging because even the most photostable fluorophores bleach within tens to hundreds of seconds under conditions required for stimulated emission depletion (STED) or SMS nanoscopy^4^.

Recently, we reported a set of high-density environmentally sensitive (HIDE) probes that support long time-lapse, single-color nanoscopy of organelles including the endoplasmic reticulum (ER), mitochondria, Golgi apparatus, and plasma membrane (PM)^5–7^. HIDE probes consist of an organelle-specific lipid or lipid-like small molecule with a reactive trans-cyclooctene (TCO) moiety and a silicon rhodamine-tetrazine (SiR-Tz)^8^ reaction partner. These two components undergo a rapid, in situ tetrazine ligation reaction^9,10^ that localizes the SiR dye at high density within the organelle membrane. In this environment, HIDE probes support the acquisition of continuous SMS and STED super-resolution movies in standard culture media (no redox chemicals) exceeding 25 mins−~50-fold longer than when the identical fluorophore is linked to an organelle-resident protein^7^. Here, we describe the development of labeling strategies and a new fluorescent dye Yale595 that enable live-cell HIDE imaging of two different organelles in two colors for extended times using STED. The ~50 nm per axis resolution achieved here significantly exceeds the ~100 nm resolution obtained when using structured illumination^11^ or lattice light-sheet microscopy^12^.

## Results

### HIDE probes assembled via SPAAC support long time-lapse live-cell nanoscopy

Two-color, live-cell HIDE nanoscopy demands not only two organelle-specific small molecules and a second photostable fluorophore but also a second conjugation reaction that is orthogonal to the tetrazine ligation and proceeds rapidly to completion within live cells. We were drawn to the strain-promoted azide-alkyne cycloaddition reaction (SPAAC)^13^ between an azide and a dibenzoazacyclooctyne (DBCO) as it is live-cell compatible and reacts rapidly enough (*k* = 0.31 M^−1^ s^−1^)^14^ to ensure complete reaction using reagents at micromolar concentrations.

**Fig. 1:**
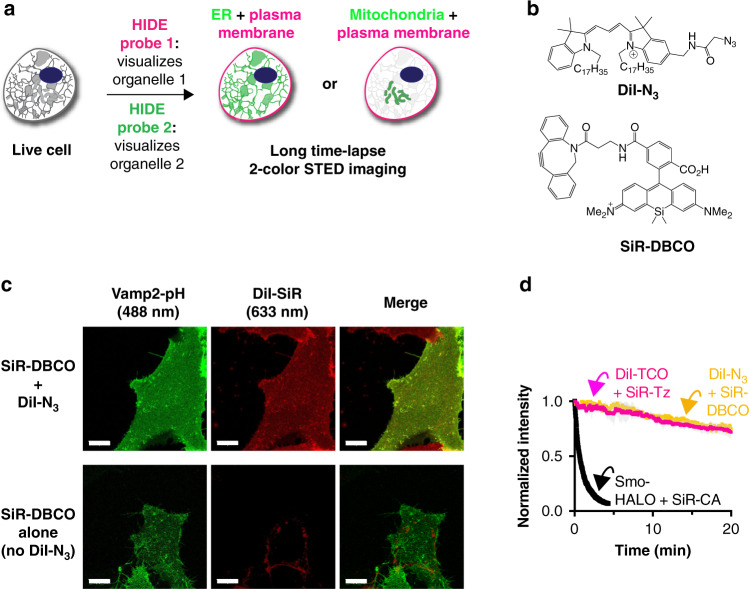
Development of two-color high-density environment-sensitive (HIDE) probes. **a** HIDE probes enable long time-lapse two-color STED imaging. **b** Chemical structures of DiI-N_3_ and SiR-DBCO. **c** Top: images of DiI-SiR (633 nm) colocalizes with plasma membrane marker Vamp2-pH (488 nm). Bottom: HeLa cells transiently expressing Vamp2-pH was incubated with 2 μm SiR-DBCO for 30 min at 37 ˚C then imaged under a spinning disk confocal microscope. When DiI-N3 was not added, SiR-DBCO (633 nm) did not colocalize with Vamp2-pH (488 nm). Scale bar: 10 μm. **d** Plot illustrating normalized fluorescence intensity of Smo-HALO + SiR-CA (black), DiI-TCO + SiR-Tz (red) and DiI-N_3_ + SiR-DBCO (yellow) over time (mean ± SD, *n* = 3 ROI, *N* = 1 cell).

To confirm that a SPAAC-assembled HIDE probe would localize correctly (Fig. 1a), DiI-N_3_ and SiR-DBCO were synthesized (Supplementary Information, Fig. 1b, Supplementary Fig. 1a), added sequentially to HeLa cells, incubated 30 min to generate DiI-SiR and imaged by confocal microscopy. Under these conditions, DiI-SiR colocalized extensively with a bona fide PM marker, VAMP2-pH (PCC = 0.63 ± 0.06); no colocalization was observed when cells were incubated with SiR-DBCO in absence of DiI-N_3_ (PCC = 0.29 ± 0.03) (Fig. 1c lower middle panel, Supplementary Fig. 2). As observed previously^5^, the HIDE probe DiI-SiR must be assembled in two steps, as cells treated with the pre-assembled reaction product DiI-SiR (Supplementary Fig. 1a) showed no PM labeling in SiR channel (Supplementary Fig. 3).

To test whether a HIDE probe assembled using SPAAC would support prolonged STED nanoscopy, we treated HeLa cells, under optimized conditions (Supplementary Fig. 4), with 10 µm DiI-N_3_ and 2 µm SiR-DBCO. We imaged the cells continuously by STED for 20 min at 0.5 Hz and monitored PM fluorescence over time (Supplementary Movie 1). Both the initial fluorescence intensity (Supplementary Fig. 5a) and bleaching half-life (Fig. 1d and Supplementary Fig. 5b) of the HIDE probe generated from DiI-N_3_ and SiR-DBCO were virtually identical to those measured using DiI-TCO and SiR-Tz (bleaching half-life 62.9 ± 1.4 min vs 65.9 ± 0.6 min). The similarity of these values confirms that HIDE probes assembled using SPAAC support prolonged STED imaging; the small difference in structure due to alternative linker chemistry has no apparent effect on SiR photostability. In contrast, STED images generated using the Smo-Halo/SiR-CA combination bleached within 1 min when visualized by STED (Fig. 1d, black trace). In addition, fluorescence recovery after photobleaching experiment support that the marked difference in photostability between Smo-Halo/SiR-CA and DiI-TCO/SiR-Tz is not due to diffusion dynamics, as we observed similar diffusion kinetics of recovery with protein and lipid labeling strategies; 10.9 ± 4.5 versus 10.3 ± 1.3 s, respectively (Supplementary Fig. 6a). Although the HIDE probes had a marginally higher mobile fraction (25%) this increase cannot account for the ~40-fold increased resistance of HIDE probes to photobleaching. As an additional control, the bleaching recovery kinetics of an independent lipid dye (Laurdan) were virtually identical in control cells to those labeled with DiI-TCO/SiR-Tz, supporting that the HIDE probe labeling causes no general changes in PM fluidity (Supplementary Fig. 6b).

### Development and evaluation of Yale595

**Fig. 2:**
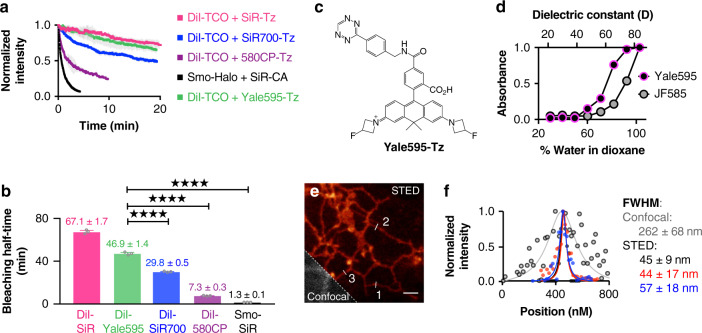
Development of Yale595. **a** Plot illustrating normalized fluorescence intensity (NI) of 580CP (purple), SiR (red), SiR700 (blue) and Yale595 (green) over time (mean ± standard deviation (SD), *n* = 3 region of interest (ROI), *N* = 1 cell). **b** Bleaching half-life calculated from a single exponential fit to the photobleaching curves in **a** (mean ± SD., *n* = 3 ROI, *N* = 1 cell). ****P ≤ 0.0001, unpaired *t* test, two-tailed. **c** Chemical structure of Yale595-Tz. **d** Plot of normalized absorbance of Yale595-COOH and JF585-COOH in response to different dielectric constant, D, of dioxane-water mixtures (mean, *n* = 2). **e** STED imaging of endoplasmic reticulum of HeLa cells labeled with 2 μm Cer-TCO for 1 hr followed by 2 μm Yale595-Tz for 30 min with a confocal cutaway in gray. Scale bar: 1 μm. **f** A plot of the fluorescence signal in the STED and confocal image as a function of position along the line profile in **e**. A fit of three line profiles from the confocal data to a Lorentzian function (gray line) provides a FWHM (mean ± SD, *n* = 3) of 262 ± 68 nm. Fits of three line profiles (white lines) in the STED image individually to a Lorentzian function provide FWHM values of 45 ± 9 nm, 44 ± 17 nm, and 57 ± 18 nm.

Next, we sought to identify a STED-appropriate fluorophore that would be compatible with SiR to enable two-color HIDE nanoscopy. The ideal second fluorophore should be: (1) membrane-permeant; (2) non-toxic; (3) highly photostable; and (4) spectrally separable from SiR. We first evaluated four previously reported fluorophores: 580CP^15^, Atto590^16,17^, JF585^18^, and SiR700^19^ (Supplementary Fig. 7). HeLa cells were treated with 10 µm DiI-TCO (Supplementary Fig. 1d) followed by 2 µm of either 580CP-Tz, Atto590-Tz, JF585-Tz, or SiR700-Tz (Supplementary Information) then imaged by STED at 0.5 Hz. None of these previously reported fluorophores were suitable. Visual inspection and time-dependent quantification of PM fluorescence revealed that the HIDE probes generated from DiI-TCO and either CP580-Tz or SiR700-Tz bleached considerably more rapidly than that generated using SiR-Tz (Fig. 2a, b, Supplementary Fig. 8; Supplementary Movie 2); further the HIDE probe generated from DiI-TCO and SiR700-Tz was also dimmer (Supplementary Fig. 8d; Supplementary Movie 3). Of note, the HIDE probe generated from DiI-TCO and Atto590-Tz was acutely cytotoxic (Supplementary Movie 4, Fig. 8a, e), whereas that generated from DiI-TCO and JF585-Tz was extremely dim (Supplementary Fig. 8b, d). Strikingly, all PM-localized HIDE probes generated here could be imaged for an order of magnitude longer then a PM-localized protein Smo-Halo/SiR-CA (Supplementary Movie 5).

We were intrigued by the poor performance of the HIDE probe generated from DiI-TCO and JF585-Tz, as this fluorophore performs well when used to label a membrane resident protein^18^. We hypothesized that this difference reflected a shift in the equilibrium between the open (ON) and closed (OFF) states of this dye in the two environments: JF585 performs well when attached to a membrane protein because it is mainly ON in a polar, aqueous environment, but poorly as a HIDE probe because it is mainly OFF in the lipid environment. A plot of JF585 emission as a function of the percent water in a water/dioxane mixture is consistent with this hypothesis; the midpoint of the transition occurs at approximately 91% water (Fig. 2d). We reasoned that a derivative of JF585 in which the 3,3-difluoroazetidine ring^20^ is replaced with a less electrophilic 3-fluoroazetidine ring to form 3-fluorazetidine carborhodamine would display a higher ON fraction in the lipid environment and thereby facilitate STED imaging. We synthesized tetrazine fluorazetidine carborhodamine (see Supplementary Information), which we named Yale595 to reflect its origin and slight red spectral maximum excitation shift (Supplementary Fig. 9). The excitation and emission spectra of Yale595-CO_2_H are compatible with SiR for two-color imaging (Supplementary Fig. 9). As anticipated, a plot of Yale595 emission as a function of the percent water in a water/dioxane mixture was shifted, with a midpoint at ~75% water (Fig. 2d). Moreover, the apparent photostabilities of HIDE probes generated from DiI-TCO and Yale595-Tz or DiI-TCO and SiR-Tz were similar (Fig. 2a, b, Supplementary Fig. 8, 10a, b and Supplementary Movie 6). To further validate the utility of Yale595 in cells, we generated an ER-specific HIDE probe from Cer-TCO^5^ and Yale595-Tz (Supplementary Fig. 1b). This probe colocalized with Sec61β-GFP (Supplementary Fig. 10c, d) and when visualized using STED could resolve individual ER tubules with a full width half-maximum (FWHM) of ~50 nm (Fig. 2e, f). Labeling with Cer-TCO and SiR-Tz was harder to visualize ER than with Yale595-Tz, which is likely due to higher nonspecific binding of SiR-Tz (Supplementary Fig. 10e, f). Per the increased photostability of Yale595 we reasoned that it results from a combination of high-density labeling enabled by the lipid probe^7^ and the low ON/OFF ratio of Yale595 in a hydrophobic membrane environment. Support for this concept derives from the observation of a comparable initial fluorescent intensity of cells whose ER membranes were labeled with Sec61β−SNAP/Yale595-BG and Cer-TCO/Yale595-Tz, but only cells that were labeled with Cer-TCO/Yale595-Tz strongly resisted photobleaching. These observations are consistent with a model in which the low ON/OFF ratio of Yale595 in the lipid environment establishes a pool of non-absorbing (dark) Yale595 molecules that can replenish absorbing (bright) Yale595 molecules that have been photo-bleached. Minimal cross-talk (<1%) between 561 nm and 595 nm channel was observed when PM-specific HIDE probe DiI-Yale595 or mitochondria-specific HIDE probe RhoB-Yale595 (Supplementary Fig. 1c) was imaged under the STED microscope (Supplementary Fig. 11). To optimize conditions to visualize cellular structures using Yale595 in live cells, we labeled HeLa cells with 10 µm DiI-TCO and 2 µm Yale595-Tz and imaged them by STED using different excitation power and STED depletion powers, as shown in Supplementary Fig. 12. These studies support that photostability was maximal when the excitation laser power (595 nm) was set to 10% and the depletion laser power (775 nm) was set to 30%; albeit the brightness was as expected higher using to 20% power. We thus used these conditions for live cells imaging experiments.

### Long time-lapse two-color live-cell nanoscopy using HIDE probes

**Fig. 3:**
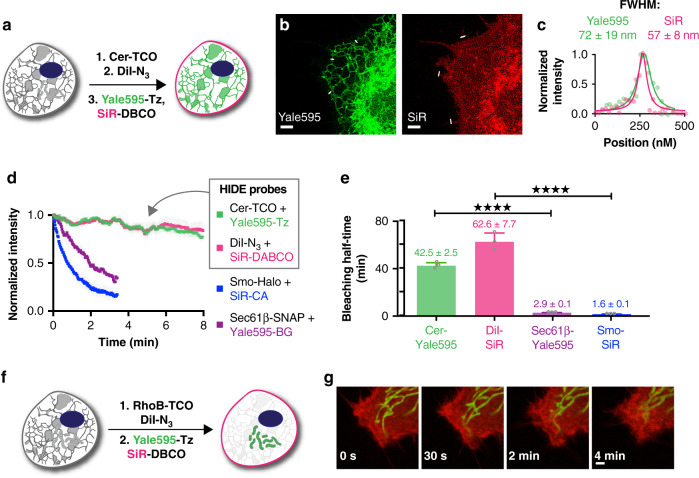
Long time-lapse two-color live-cell STED imaging of HeLa cells. **a** Schematic illustration of the three-step procedure employed to label the plasma membrane and ER. **b** Two-color STED image of the plasma membrane and ER of HeLa cells. Images from both channels are shown. Scale bars: 2 μm. **c** A plot of the fluorescence signal in the two-color STED image as a function of position along the line profile in **b**. A fit of the line profile from the Yale595 channel to a Lorentzian function (green line) provides a FWHM of 72 ± 19 nm. A fit of the line profile from the SiR channel to a Lorentzian function (red line) provides a FWHM of 57 ± 8 nm. **d** Plot of normalized fluorescence intensity of protein tags and HIDE probes over time (mean ± standard deviation (SD), *n* = 3 region of interest (ROI)). The fluorescence intensity was measured in each channel separately. **e** Bleaching half-life calculated from a single exponential fit to the photobleaching curves in **d** (mean ± SD, *n* = 3 ROI). ****P ≤ 0.0001, unpaired *t* test, two-tailed. **f** Schematic illustration of the two-step procedure employed to label the plasma membrane and mitochondria. **g** Time course images of the plasma membrane and mitochondria. Scale bar: 2 μm.

The ER HIDE probe generated using Yale595-Tz was then used in combination with DiI-N_3_/SiR-DBCO to achieve two-color HIDE imaging of the ER and PM. HeLa cells were treated first with DiI-N_3_ and Cer-TCO then with SiR-DBCO and Yale595-Tz (Fig. 3a, Supplementary Fig. 13) and imaged by STED (Supplementary Fig. 3b). High-resolution images were obtained in both channels (FWHM = 72 ± 19 nm for Yale 595; 57 ± 8 nm for SiR (Fig. 3c) with cross-talk between the two channels minimized by spectral unmixing (Supplementary Fig. 14). No cross-reactivity was observed when HeLa cells were labeled with Cer-TCO + Yale595-DBCO or DiI-N_3_ + Yale595-Tz (Supplementary Fig. 7c, Supplementary Fig. 15). With the two-color HIDE probes, we observed ER tubule formation, elongation, and rearrangement as well as filopodia movement over 250 time points with no significant loss of fluorescence (Fig. 3d, e, Supplementary Movie 7). When conjugated to ER (Sec61β)- and PM (Smo)-resident proteins through Halo and SNAP tags, respectively, SiR and Yale595 bleached 15- to 40-fold faster (Fig. 3d, e, Supplementary Movie 8). The two-color HIDE strategy was easily generalized to simultaneously image the PM and mitochondria in live cells for extended times (Fig. 3f, g). Namely, HeLa cells were treated with DiI-N_3_ along with the mitochondria-specific small molecule RhoB-TCO followed by SiR-DBCO and Yale595-Tz (Fig. 3f, Supplementary Fig. 16) then imaged by STED at 0.5 Hz (Fig. 3g, Supplementary Movie 9). Here, the fine and rapid dynamic processes of mitochondria fission and filopodia remodeling could be observed and studied over 5 min with minimal photobleaching. The toxicity of two-color HIDE probes was studied by monitoring cell division events of HeLa cells. No toxicity of the probes was observed, as monitored by the number of cell division events per hour after probe treatment (Supplementary Fig. 17).

**Fig. 4:**
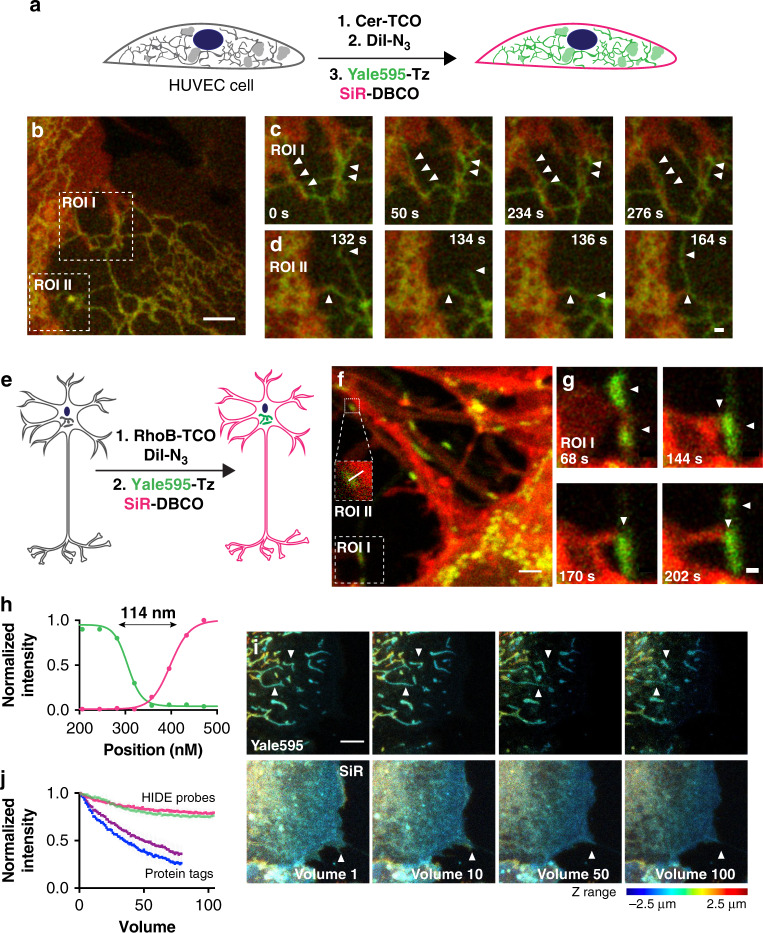
Application of two-color HIDE probes to primary cell lines. **a** Schematic illustration of the three-step procedure employed to label the plasma membrane and ER of Human umbilical vein endothelial cells (HUVECs). **b** Snapshot of a two-color STED movie of HUVEC. Scale bar: 2 μm. **c**, **d** Time-lapse images of ER dynamics and interactions between filopodia and ER. Scale bars: 500 nm. **e** Schematic illustration of the two-step procedure employed to label the plasma membrane and mitochondria of DIV4 mouse hippocampal neurons. **f** Snapshot of a two-color STED movie of DIV4 mouse hippocampal neurons. Scale bar: 2 μm. **g** Time-lapse images of interactions between filopodia and mitochondria. Scale bars: 500 nm. **h** Plot of line profile shown in (**f**, ROI II) illustrating the distance between plasma membrane and mitochondria. **i** Time-lapse two-color confocal imaging of mitochondria and plasma membrane in retinal pigment epithelium (RPE) cells. The mitochondrial and plasma membrane volumetric dynamics are recorded continuously over seconds. The axial information is color-coded. Twenty z-stacks per volume. volume rate: 6.1 s. Scale bar: 2 μm. **j** Plot illustrating normalized fluorescence intensity of RhoB-Yale595 (green), DiI-SiR (red), SMO25-Yale595 (purple), and Smo-SiR (blue) over time (mean ± standard deviation, *n* = 3 region of interest, *N* = 1 cell).

As HIDE probes are generated from pairs of cell-permeant small molecules, they can be used to label both primary and hard-to-transfect cells^6^. To highlight this versatility, we imaged pairs of organelles in two colors by STED in three types of primary cells: HUVEC, mouse hippocampal neurons, and retinal pigment epithelium (RPE) cells (Fig. 4). Two-color images of the PM and ER of HUVEC cells with Cer-TCO/Yale595-Tz and DiI-N_3_/SiR-DBCO revealed filopodia of one cell strikingly proximal to the ER of an adjacent cell (see ROI I and II in Fig. 4b, c, Supplementary Movie 10, for two more examples, see Supplementary Movies 12, 13). This interaction was observed in 13 of the 15 HUVECs imaged. These interactions persisted for several minutes (Fig. 4b, arrows). To quantify the number of apparent ER–PM interactions in each movie we counted the number of long-term ER–PM interactions that persisted throughout each movie. To rule out these being random colocalization we compared them with the long-term ER–PM interactions that persisted throughout each movie when the 595 nm channel was flipped 180° (Supplemental Fig. 18). We observed significant higher number of events in the former case, supporting that the apparent ER–PM interactions that we saw is not stochastic. Interestingly, although the ER in a single cell is known to form contacts with the PM^21^, the inter-cell interactions evident here have to our knowledge previously not been observed and may potentially represent a new site of inter-cellular communication, an area of wide general interest^22^. Aside, structure such as tunneling nanotubes while now well accepted in many cell as important 50–200 nm thin tunnels between cells were only relatively recently discovered via serial EM^23^—highlighting the link between advanced imaging and detection of new interaction. In another example, mouse hippocampal neurons were labeled with the dual HIDE PM and mitochondria probes and imaged by STED (Fig. 4e, f). We can discern two separate structures, dendritic membrane and mitochondria, only 114 nm apart (Fig. 4f, ROI II, Fig. 4h). We also observed interactions between dendritic filopodia and mitochondria over a few minutes (Fig. 4g, Supplementary Movie 11).

Importantly, the benefits of two-component HIDE probes are not limited to STED. A common photon-demanding imaging modality is 3D time-lapse (4D) imaging. To test the benefit of dual color HIDE probes, we compared conventional confocal 4D imaging of dual HIDE probes to cells tagged with the protein markers SMO25-Yale595 (mitochondria) and Smo-SiR (PM). Here too there was a major benefit of imaging with HIDE probes as SiR and Yale595 tagged HIDE probes were up to eight times more photostable than the comparable protein tags (Fig. 4i, j).

The HIDE probes developed here enabled two-color time-lapse live-cell STED imaging for hundreds of time points with virtually no bleaching, far exceeding the duration using state-of-the-art protein tags by over an order of magnitude. The labeling protocols can easily be adapted to various cell lines, including hard-to-transfect and primary cells. We envision that imaging experiments that require a large photon budget would generally benefit from these densely labeled and photostable organelle HIDE probes.

## Methods

### Chemical synthesis

Synthesis protocol and additional characterization can be found in the Supplementary Methods.

### Cell culture

HeLa cells (ATCC) were cultured in Dulbecco’s modified Eagle medium (DMEM) (Gibco) supplemented with 10% fetal bovine serum (FBS) (Sigma-Aldrich), penicillin (100 unit/mL) and streptomycin (100 μg/mL). hTERT RPE-1 cells (ATCC, CRT-400) were cultured in DMEM/F12 (Gibco) supplemented with 10% FBS, 1% nonessential amino acids (Gibco), 2 mm sodium pyruvate (Gibco), penicillin (100 unit/mL) and streptomycin (100 μg/mL). Human umbilical vein endothelial cells (HUVEC) (Lonza, C2517A) were cultured in EGMTM-2 Endothelial Cell Growth Medium-2 BulletKit (Lonza, CC-3162). All cells were purchased from commercial sources and periodically tested for mycoplasma with DNA methods. Primary neurons were isolated from P0 to P1 mouse hippocampus using papain digestion and plated in Neurobasal A media (Invitrogen) with 2% Gem21 supplement (Gemini) and 10% FBS on glass-bottom culture dishes (Matek) coated with poly-d-lysine (20 μg/mL, Corning) and laminin 111 (1 μg/mL, Corning). Media was changed on the neurons after 4 h and maintained in serum-free Neurobasal A/Gem21 media containing 1% pen/strep and 2 mm l-glutamine.

### Plasmid

The SNAP-Tag plasmid Sec61β-SNAP and OMP25-SNAP was obtained from the Rothman lab and the Bewersdorf lab at Yale School of Medicine^16^.

pLVX-ss-HaloTag-Smo: the ss-HaloTag-Smo fragment was PCR amplified from pC4S1-ss-Halotag-mSmo^7^ and cloned by In-Fusion HD into pLVX-puro digested with *Eco*RI and *Bam*HI to generate pLVX-ss-HaloTag-Smo for lentiviral production.

### Confocal microscopy

Spinning disk confocal microscopy was performed using an Improvision UltraVIEW VoX system (Perkin-Elmer) built around a Olympus XI71 inverted microscope, equipped with PlanApo objectives (×60, 1.45 NA) and controlled by the Volocity software (Improvision). To image DiI and SiR, 561-nm and 640-nm laser lines with appropriate filters (615 ± 35 and 705 ± 45 nm, respectively) were used.

### STED microscopy

Live-cell STED microscopy was performed on a Leica TCS SP8 Gated STED ×3 microscope. The microscope is equipped with a tunable (460–660 nm) pulsed white light laser for excitation and two HyD detectors for tunable spectral detection. There are also two additional PMT detectors. The microscope is outfitted with three STED depletion lasers (592 nm, 660 nm, and 775 nm). For live-cell imaging, the microscope was equipped with a Tokai Hit stage top incubator (model: INUBG2A-GSI) with temperature and CO_2_ control to maintain an environment of 37 °C and 5% CO_2_. Detailed information on excitation laser and detection window varies depend on the dyes and protocols used and is indicated below in each section. Imaging was conducted with a ×100 oil immersion objective (HC PL APO ×100/1.40 oil) at 1000 Hz with two line accumulations in a 19.38 µm^2^ field of view (1024 × 1024 pixels at 18.94 nm/pixel). Raw microscopy data were Gaussian blurred (2.0 pixels) in ImageJ. The FWHM values were obtained by fitting line profiles to a Lorentz distribution using Origin 9.1 (www.originlab.com).

### Labeling of PM with DiI-N_3_ and SiR-DBCO

HeLa cells were incubated with 500 μL of 10 μm DiI-N_3_ in phosphate-buffered saline (PBS) containing 1% casein hydrolysate for 3 min at 37 °C. The cells were then washed and incubated with 500 μL of 2 μm SiR-DBCO in PBS containing 1% casein hydrolysate for 30 min at 37 °C. After washing, DMEM ph(−) was added as imaging buffer, and confocal imaging was performed at 37 °C.

### Optimization of labeling conditions

First, the concentration of DiI-N_3_ was optimized. HeLa cells were incubated with 500 μL of 5 μm, 10 μm, 15 μm, or 20 μm in PBS containing 1% casein hydrolysate DiI-N_3_ for 3 min at 37 °C. After washing, DMEM ph(−) was added as imaging buffer, and confocal imaging was performed at 37 °C. Then the concentration of SiR-DBCO was optimized. HeLa cells were incubated with 500 μL of 10 μm DiI-N_3_ in PBS containing 1% casein hydrolysate for 3 min at 37 °C. The cells were then washed and incubated with 500 μL of 2 μm, 5 μm, or 10 μm SiR-DBCO in PBS containing 1% casein hydrolysate for 30 min at 37 °C. After washing, DMEM ph(−) was added as imaging buffer, and confocal imaging was performed at 37 °C.

### Labeling with pre-mixed DiI-N_3_ and SiR-DBCO

In all, 500 μL of 10 μm DiI-N_3_ and 2 μm, 5 μm, or 10 μm SiR-DBCO in PBS containing 1% casein hydrolysate was incubated at 37 °C for 1 h. The formation of the product was confirmed by MS analysis. The resulting mixture was added to HeLa cells and incubated for 3 min at 37 °C. After washing, DMEM ph(−) was added as imaging buffer, and confocal imaging was performed at 37 °C.

### Photostability of DiI-N_3_ and SiR-DBCO

HeLa cells were labeled as described in “Labeling of plasma membrane with DiI-N_3_ and SiR-DBCO” and imaged under STED microscope at 37 °C. SiR dye was excited at 633 nm (40% power) and their emission was detected using a HyD detector from 650 to 737 nm. The 775 nm depletion laser was used for STED microscopy (30% power). Images were recorded continuously at 2 s per frame. Photobleaching plots were generated by normalizing background-subtracted fluorescence intensities (PM ROI−background ROI) from three separate ROIs using the ROI manager Multi Measure tool in ImageJ.

### Photostability of 580CP, JF585, Atto590 and SiR700

HeLa cells were incubated with 500 μL of 10 μm DiI-TCO in PBS containing 1% casein hydrolysate for 3 min at 37 °C. The cells were then washed and incubated with 500 μL of 2 μm 580CP-Tz, JF585-Tz, Atto590-Tz or SiR700 in PBS containing 1% casein hydrolysate for 30 min at 37 °C. After washing, DMEM ph(−)was added as imaging buffer, and STED imaging was performed at 37 °C. 580CP, and Atto590 were excited at 590 nm (5% power) and their emission was detected using a HyD detector from 600 to 670 nm. JF585 dye was excited at 590 nm (15% power) and their emission was detected using a HyD detector from 600–670 nm. SiR700 dye was excited at 650 nm (40% power) and their emission was detected using a HyD detector from 660 to 730 nm. The 775 nm depletion laser was used for STED microscopy (30% power). Images were recorded continuously at 2 s per frame. Photobleaching plots were generated by normalizing background-subtracted fluorescence intensities (PM ROI−background ROI) from three separate ROIs using the ROI manager Multi Measure tool in ImageJ.

### Absorption and emission spectra of Yale 595 and SiR

The absorbance spectra of Yale595 and SiR were measured at 2 μm in Dulbecco’s phosphate-buffered saline (DPBS, w/ 0.1% dimethyl sulfoxide; DMSO) on a Beckman UV-Vis spectrophotomerter. The emission spectra of the same solutions were measured on a TCSPC TD-Fluor Horiba Fluorolog 3 Time Domain Fluorimeter.

### Quantum yield for Yale595

Solutions of Yale595 and Bodipy-Texas Red between 0 and 10 μm were prepared in DPBS (w/ 0.05–0.25% DMSO). Absorbance at 550 nm was measured for each on a Beckman UV-Vis spectrophotomerter. Afterwards, fluorescence of the same samples was measured on a TCSPC TD-Fluor Horiba Fluorolog 3 Time Domain Fluorimeter. Both the excitation and emission bandwidths for all measurements were set to 5 nm. The spectra were recorded with a step size of 1 nm. The excitation wavelength was set to 550 nm and the emission spectra were recorded in the 560–800 nm interval. Absorbance at 550 nm versus integrated fluorescence intensity for each solution was plotted using GraphPad Prism 7.0. The relative quantum yield of Yale595 was calculated from the linear regressions according to the following:

1 $$\begin{array}{*{20}{c}} {QY_{exp} = \frac{{m_{exp}}}{{m_{std}}}QY_{std}\ } \end{array}$$

Where *QY*_*exp*_ and *m*_*exp*_ are the relative quantum yield and slope of linear regression, respectively, for Yale595 and *QY*_*std*_ and *m*_*std*_ are the absolute quantum yield and slope of linear regression, respectively, for Bodipy-Texas Red. The absolute quantum yield for Bodipy-Texas Red was previously reported.

### Extinction coefficient for Yale595

Solutions of Yale595 and SiR at 5 μm, 10 μm, 15 μm, 20 μm, and 25 μm in DPBS (w/ 0.25–1.25% DMSO) were prepared. The absorbance at 597 nm (Yale595) or 652 nm (SiR) were measured on Spiegel Lab plate reader using polypropylene 96-well plates. The concentrations were plotted against their absorbances using GraphPad Prism 7.0. The extinction coefficient of Yale595 was calculated from the linear regressions according to the following:

2 $$\begin{array}{*{20}{c}} {\varepsilon _{exp} = \frac{{m_{exp}}}{{m_{std}}}\varepsilon _{std}\ } \end{array}$$

Where *ε*_*exp*_ and *m*_*exp*_ are the extinction coefficient and slope of linear regression, respectively, for Yale595 and *ε*_*std*_ and *m*_*std*_ are the extinction coefficient and slope of linear regression, respectively, for SiR. The extinction coefficient for SiR was previously reported^8^.

### Absorbance in response to different solvent polarities

Yale595-COOH (or JF585-COOH) was dissolved into a mixture of water and dioxane (0–100% dioxane in water) to a final concentration of 10 μm. The absorbance data was recorded on a DU730 Life Science UV/Vis spectrophotometer using the wavelength scan mode.

### Photostability of Yale595

HeLa cells were incubated with 500 μL of 10 μm DiI-TCO in PBS containing 1% casein hydrolysate for 3 min at 37 °C. The cells were then washed and incubated with 500 μL of 2 μm Yale595-Tz in PBS containing 1% casein hydrolysate for 30 min at 37 °C. After washing, DMEM ph(−)was added as imaging buffer, and STED imaging was performed at 37 °C. Yale595 dye was excited at 595 nm (10% power) and their emission was detected using a HyD detector from 605–675 nm. The 775 nm depletion laser was used for STED microscopy (30% power). Images were recorded continuously at 2 s per frame. Photobleaching plots were generated by normalizing background-subtracted fluorescence intensities (PM ROI–background ROI) from three separate ROIs using the ROI manager Multi Measure tool in ImageJ.

### Live-cell STED imaging of ER using Yale595

HeLa cells were incubated with 500 μL of 2 μm Cer-TCO in PBS containing 1% casein hydrolysate and 0.2% Pluronic F-127 for 60 min at 37 °C. The cells were then washed and incubated with 500 μL of 2 μm Yale595-Tz in PBS containing 1% casein hydrolysate for 30 min at 37 °C and imaged on the SP8 STED microscope. Yale595 dye was excited at 595 nm (10% power) and their emission was detected using a HyD detector from 605–675 nm. The 775 nm depletion laser was used for STED microscopy (30% power).

### Labeling of PM and ER of HeLa cells

HeLa cells were incubated with 500 μL of 4 μm Cer-TCO in Live Cell Imaging Solution (Life Technologies, A14291DJ) containing 0.2% Pluronic F-127 for 60 min at 37 °C. After washing, the cells were treated with 500 μL of 10 μm Dil-N_3_ in Live Cell Imaging Solution for 3 min at 37 °C. After washing, the cells were incubated with 500 μL of 2 μm SiR-DBCO and 2 μm Yale595-Tz in Live Cell Imaging Solution for 30 min at 37 °C. After washing, STED imaging was carried out at 37 °C using DMEM ph(−) as the imaging buffer under Leica SP8 microscope with two excitation lasers at 595 nm (10% power), 650 nm (60% power) and two detection windows at 605–625 nm and 670–750 nm. The 775 nm depletion laser was used for STED microscopy (30% power). Images were recorded continuously at 2 s per frame. Raw image data were spectral unmixed and Gaussian blurred (2.0 pixels) in ImageJ.

### Labeling of PM and mitochondria of HeLa cells

HeLa cells were incubated with 500 μL of 10 μm RhoB-TCO and 10 μm Dil-N_3_ in Live Cell Imaging Solution for 3 min at 37 °C. After washing, the cells were incubated with 500 μL of 2 μm SiR-DBCO and 2 μm Yale595-Tz in Live Cell Imaging Solution for 30 min at 37 °C. After washing, cells were incubated at 37 °C in DMEM ph(−) for 30 min to wash out the remaining dyes. After further washing, STED imaging was carried out at 37 °C using DMEM ph(−) as the imaging buffer under Leica SP8 microscope with two excitation lasers at 595 nm (10% power), 650 nm (60% power) and two detection windows at 605–625 nm and 670–750 nm. The 775 nm depletion laser was used for STED microscopy (30% power). Images were recorded continuously at 2 s per frame. Raw image data were spectral unmixed and Gaussian blurred (2.0 pixels) in ImageJ.

### Labeling of PM and ER of HUVEC

HUVECs were incubated with 500 μL of 4 μm Cer-TCO in Live Cell Imaging Solution containing 0.2% Pluronic F-127 for 60 min at 37 °C. After washing, the cells were treated with 500 μL of 10 μm Dil-N_3_ in Live Cell Imaging Solution for 3 min at 37 °C. After washing, the cells were incubated with 500 μL of 2 μm SiR-DBCO and 2 μm Yale595-Tz in Live Cell Imaging Solution for 30 min at 37 °C. After washing, STED imaging was carried out at 37 °C using DMEM ph(−) as the imaging buffer under Leica SP8 microscope with two excitation lasers at 594 nm (10% power), 650 nm (60% power) and two detection windows at 605–625 nm and 670–750 nm. The 775 nm depletion laser was used for STED microscopy (30% power). Images were recorded continuously at 2 s per frame. Raw image data were spectral unmixed and Gaussian blurred (2.0 pixels) in ImageJ.

### Labeling of PM and mitochondria of neurons

Neuron cells were incubated with 500 μL of 10 μm RhoB-TCO and 10 μm Dil-N_3_ in DMEM for 3 min at 37 °C. After washing, the cells were incubated with 500 μL of 2 μm SiR-DBCO and 2 μm Yale595-Tz in DMEM for 30 min at 37 °C. After washing, cells were incubated at 37 °C in DMEM ph(−) for 30 min to wash out the remaining dyes. After further washing, STED imaging was carried out at 37 °C using DMEM as the imaging buffer under Leica SP8 microscope with two excitation lasers at 595 nm (10% power), 650 nm (60% power) and two detection windows at 605–625 nm and 670–750 nm. The 775 nm depletion laser was used for STED microscopy (30% power). Images were recorded continuously at 2 s per frame. Raw image data were Gaussian blurred (2.0 pixels) in ImageJ.

### Labeling of PM and ER with protein tags

Generation of hTERT RPE-1 cells stably expressing Smo-Halo was described previously using pLVX-ss-HaloTag-Smo^24^. hTERT RPE-1 cells stably expressing Smo-Halo were transfected with Sec61β-SNAP using FuGENE HD Transfection Reagent (Promega) one day before imaging. Before imaging, the cells were incubated with 500 μL of 2 μm SiR-CA and 2 μm Yale595-BG in Live Cell Imaging Buffer for 30 min at 37 °C. After washing, STED imaging was carried out at 37 °C using DMEM as the imaging buffer under Leica SP8 microscope with two excitation lasers at 595 nm (10% power), 650 nm (60% power) and two detection windows at 605–625 nm and 670–750 nm. The 775 nm depletion laser was used for STED microscopy (30% power). Images were recorded continuously at 2 s per frame. Raw image data were spectral unmixed Gaussian blurred (2.0 pixels) in ImageJ.

### Labeling of PM and mitochondria with protein tags

hTERT RPE-1 cells stably expressing Smo-Halo were transfected with OMP25-SNAP using FuGENE HD Transfection Reagent (Promega) one day before imaging. Before imaging, the cells were incubated with 500 μL of 2 μm SiR-CA and 2 μm Yale595-BG in Live Cell Imaging Buffer for 30 min at 37 °C. After washing, STED imaging was carried out at 37 °C using DMEM as the imaging buffer under Leica SP8 microscope with two excitation lasers at 595 nm (10% power), 650 nm (60% power) and two detection windows at 605–625 nm and 670–750 nm. The 775 nm depletion laser was used for STED microscopy (30% power). Images were recorded continuously at 2 s per frame. Raw image data were spectral unmixed Gaussian blurred (2.0 pixels) in ImageJ.

### Live-cell two-color 3D confocal imaging using HIDE probes

HeLa cells were incubated with 500 μL of 10 μm RhoB-TCO and 10 μm Dil-N_3_ in Live Cell Imaging Solution for 3 min at 37 °C. After washing, the cells were incubated with 500 μL of 2 μm SiR-DBCO and 2 μm Yale595-Tz in Live Cell Imaging Solution for 30 min at 37 °C. After washing, cells were incubated at 37 °C in DMEM ph(−) for 30 min to wash out the remaining dyes. After further washing, 3D confocal imaging was carried out at 37 °C using DMEM ph(−) as the imaging buffer under Leica SP8 microscope with two excitation lasers at 595 nm (3% power), 650 nm (20% power) and two detection windows at 605–625 nm and 670–750 nm. The images were taken at 20 z-stacks per volume at 6.1 s per volume. Raw image data were spectral unmixed in ImageJ.

### Evaluation of cellular toxicity of HIDE probes^6^

We monitored the cell division in three groups of HeLa cells. (1) non-treated cells; 2) HeLa cells labeled as described in 5.1; and 3) HeLa cells labeled as described in 5.2. Labeling of the cells was confirmed by a confocal microscopy. Wide field imaging was carried out on an Invitrogen EVOS FL Auto 2 Imaging system equipped with temperature, humidity, and atmosphere controls for live-cell imaging. The sample was kept at 37 °C with 5% CO_2_ and imaged using a ×20 objective and a high sensitivity 1.3 MP CMOS camera (1328 × 1048 pixels) every 10 min for 15 h using bright field. Cell division events were counted manually.

### Reporting summary

Further information on research design is available in the Nature Research Reporting Summary linked to this article.

## Supplementary information

Supplementary Information

Description of Additional Supplementary Files

Supplementary Movie 1

Supplementary Movie 2

Supplementary Movie 3

Supplementary Movie 4

Supplementary Movie 5

Supplementary Movie 6

Supplementary Movie 7

Supplementary Movie 8

Supplementary Movie 9

Supplementary Movie 10

Supplementary Movie 11

Supplementary Movie 12

Supplementary Movie 13

Reporting Summary

## Data Availability

All the data supporting this study are available within the article, the Supplementary file, and from the corresponding authors upon reasonable request, as indicated in the Reporting Summary for this article.

## References

[1] Abbe E (1873). Beiträge zur Theorie des Mikroskops und der mikroskopischen Wahrnehmung. Arch. Mikrosk. Anat..

[2] Sahl SJ, Hell SW, Jakobs S (2017). Fluorescence nanoscopy in cell biology. Nat. Rev. Mol. Cell Biol..

[3] Nixon-Abell, J. et al. Increased spatiotemporal resolution reveals highly dynamic dense tubular matrices in the peripheral ER. *Science* **354**, aaf3928(2016). 10.1126/science.aaf3928 PMC6528812 27789813

[4] Oracz, J., Westphal, V., Radzewicz, C., Sahl, S. J. & Hell, S. W. Photobleaching in STED nanoscopy and its dependence on the photon flux applied for reversible silencing of the fluorophore. *Sci. Rep.* **7**, 11354(2017). 10.1038/s41598-017-09902-x PMC5595794 28900102

[5] Erdmann RS (2014). Super-resolution imaging of the golgi in live cells with a bioorthogonal ceramide probe. Angew. Chem. Int. Ed..

[6] Thompson AD (2017). Long-term live-cell STED nanoscopy of primary and cultured cells with the plasma membrane HIDE probe DiI-SiR. Angew. Chem. Int Ed..

[7] Takakura H (2017). Long time-lapse nanoscopy with spontaneously blinking membrane probes. Nat. Biotechnol..

[8] Lukinavicius G (2013). A near-infrared fluorophore for live-cell super-resolution microscopy of cellular proteins. Nat. Chem..

[9] Blackman ML, Royzen M, Fox JM (2008). Tetrazine ligation: fast bioconjugation based on inverse-electron-demand Diels-Alder reactivity. J. Am. Chem. Soc..

[10] Devaraj NK, Weissleder R, Hilderbrand SA (2008). Tetrazine-based cycloadditions: application to pretargeted live cell imaging. Bioconjugate Chem..

[11] Han, Y. B., Li, M. H., Qiu, F. W., Zhang, M. & Zhang, Y. H. Cell-permeable organic fluorescent probes for live-cell long-term super-resolution imaging reveal lysosome-mitochondrion interactions. *Nat. Commun.* **8**, 1307 (2017). 10.1038/s41467-017-01503-6 PMC5670236 29101340

[12] Chen BC (2014). Lattice light-sheet microscopy: imaging molecules to embryos at high spatiotemporal resolution. Science.

[13] Agard NJ, Prescher JA, Bertozzi CR (2005). A strain-promoted [3+2] azide-alkyne cycloaddition for covalent modification of biomolecules in living systems (vol 126, pg 15046, 2004). J. Am. Chem. Soc..

[14] Debets MF (2010). Aza-dibenzocyclooctynes for fast and efficient enzyme PEGylation via copper-free (3+2) cycloaddition. Chem. Commun..

[15] Butkevich AN (2016). Fluorescent Rhodamines and Fluorogenic Carbopyronines for Super-Resolution STED Microscopy in Living Cells. Angew. Chem. Int Ed..

[16] Bottanelli, F. et al. Two-colour live-cell nanoscale imaging of intracellular targets. *Nat. Commun.* **7**, 10778 (2016). 10.1038/ncomms10778 PMC4785223 26940217

[17] Neumann D, Buckers J, Kastrup L, Hell SW, Jakobs S (2010). Two-color STED microscopy reveals different degrees of colocalization between hexokinase-I and the three human VDAC isoforms. PMC Biophys..

[18] Grimm JB (2017). A general method to fine-tune fluorophores for live-cell and in vivo imaging. Nat. Methods.

[19] Lukinavicius G (2016). Fluorogenic probes for multicolor imaging in living cells. J. Am. Chem. Soc..

[20] Grimm, J. B. et al. A general method to improve fluorophores for live-cell and single-molecule microscopy. *Nat. Methods* **12**, 244–250 (2015). 10.1038/nmeth.3256 PMC4344395 25599551

[21] Phillips MJ, Voeltz GK (2016). Structure and function of ER membrane contact sites with other organelles. Nat. Rev. Mol. Cell Biol..

[22] Murray LMA, Krasnodembskaya AD (2019). Concise review: intercellular communication via organelle transfer in the biology and therapeutic applications of stem cells. Stem Cells.

[23] Rustom A, Saffrich R, Markovic I, Walther P, Gerdes HH (2004). Nanotubular highways for intercellular organelle transport. Science.

[24] Kukic I, Rivera-Molina F, Toomre D (2016). The IN/OUT assay: a new tool to study ciliogenesis. Cilia.

