# Supplementary material for: Two-color nanoscopy of organelles for extended times with HIDE probes
Source: Nat Commun. 2020 Aug 26;11:4271. doi: 10.1038/s41467-020-17859-1 (PMC7450044; doi:10.1038/s41467-020-17859-1)
Supplement: Supplementary file 1 — Supplementary Information [file 41467_2020_17859_MOESM1_ESM.pdf]

Supplementary Information

Two-color nanoscopy of organelles for extended times with HIDE probes

Chu et al.

Supplementary Methods

Chemical Synthesis

A. General considerations. Chemicals used for synthesis were purchased from commercial sources and were used without further purification. Flash chromatography was performed using a Teledyne Isco CombiFlash Rf using pre-packed columns with RediSep Rf silica (40–60 μm). ^{1}H NMR and ^{13}C NMR spectra were recorded on an Agilent DD2 600 NMR spectrometer (600 MHz for ^{1}H and 151 MHz for ^{13}C). The values of chemical shifts (δ) are reported in p.p.m. relative to the solvent residual signals of CD_3OD (3.31 p.p.m. for ^{1}H, 49.00 p.p.m. for ^{13}C), CD_2Cl_2 (5.33 p.p.m. for ^{1}H, 53.84 p.p.m. for ^{13}C) or CD_3CN (1.94 p.p.m. for ^{1}H, 1.32, 118.26 p.p.m. for ^{13}C). Coupling constants (*J*) are reported in Hz. High-resolution mass spectra (HRMS) were recorded on a Waters Xevo QTOF LCMS with ESI using a Waters Acquity UPLC. HPLC purifications were performed on a reverse-phase colμMn (GL Sciences, Inertsil ODS-3 10 mm × 250 mm and Inertsil ODS-3 20 mm × 250 mm) using a HPLC system composed of a pump (Jasco PU-2080 or PU-2087) and a detector (Jasco MD-2010 or MD-2018).

B. Experimental. Cer-TCO[1], DiI-TCO[2], RhoB-TCO[2], Atto590-Tz[3], and SiR-Tz[4] were synthesized as described previously.

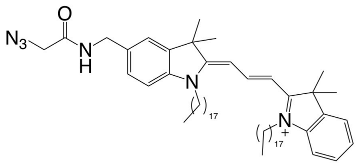

Synthesis of DiI-N_3 1. DiI-NH_2 (10 mg, 1.0 eq), azido-acetic acid-NHS (Sigma-Aldrich, 1.2 eq) and DIPEA (3.0 eq) were dissolved in DMF (1 mL) under an argon atmosphere, stirred for 6 h, and concentrated under reduced pressure. The residue was subjected to flash column chromatography (100:0 CH_2Cl_2:CH_3OH / CH_2Cl_2:CH_3OH 90:10) to yield DiI-N_3 as a pink solid (90% yield).

^{1}H NMR (600 MHz, Methanol-*d*$_4$) δ 8.57 (dd, *J* = 13.4, 13.4 Hz, 1H), 7.58 (d, *J* = 7.4 Hz, 1H), 7.53 (s, 1H), 7.48 (dd, *J* = 7.7, 7.7 Hz, 1H), 7.43 (d, *J* = 8.1 Hz, 1H), 7.38 (d, *J* = 8.0 Hz, 1H), 7.35 (dd, *J* = 8.8, 8.8 Hz, 2H), 6.49 (d, *J* = 13.5 Hz, 1H), 6.48 (d, *J* = 13.4 Hz, 1H), 4.51 (s, 2H), 4.24 – 4.14 (m, 4H), 3.98 (s, 2H), 1.91 – 1.83 (m, 4H), 1.80 (s, 12H), 1.58 – 1.22 (m, 60H), 0.92 (t, *J* = 6.9 Hz, 6H).

^{13}C NMR (151 MHz, CD_3OD) δ 176.87, 176.78, 171.03, 152.78, 144.21, 143.54, 143.40, 143.08, 138.59, 130.87, 130.34, 127.67, 124.40, 123.92, 113.36, 113.30, 104.65, 104.62, 53.92, 51.53, 51.46, 46.15, 46.10, 44.70, 33.95, 31.66, 31.64, 31.61, 31.49, 31.47, 31.46, 31.45, 31.36, 31.28, 29.39, 29.38, 29.19, 28.64, 28.64, 24.62, 15.34.

HRMS [$C_{62}H_{101}N_6O^+$] Cal: 945.8031, Obs: 945.8050.

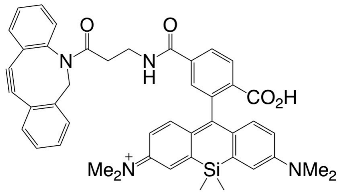

Synthesis of SiR-DBCO 2. SiR-COOH (5 mg, 1 eq.)[4], TSTU (1.2 eq.) and DIPEA (2 eq.) were dissolved in DMSO and stirred at room temperature for 20 min. DBCO-NH_2 (Click Chemistry Tools LLC, 1.2 eq.) and DIPEA (2 eq.) was added and the stirring was continued for 2 hours until LC-MS showed no remaining starting material. The mixture was then filtered and purified by reverse phase HPLC using eluent A (H_2O with 0.1% TFA) and eluent B (CH3CN with 0.1% TFA) to give SiR-DBCO as a blue solid (64% yield).

^{1}H NMR (600 MHz, Methanol-d_4) δ 8.21 (d, 1H, *J* = 8.0 Hz), 8.03 (d, 1H, *J* = 8.0 Hz), 7.86 (s, 1H), 7.72 (d, *J* = 6.4 Hz, 1H), 7.60-7.40 (m, 5H), 7.18-7.03 (m, 4H), 6.74 (d, 2H, *J* = 9.2 Hz), 6.67 (dd, 2H, *J* = 9.0, 3.0 Hz), 5.87 (br s, 1H), 5.16 (d, *J* = 13.8 Hz, 1H), 3.65-3.58 (m, 4H), 2.98 (s, 12 H), 0.78 (s, 3 H), 0.68 (s, 3 H).

^{13}C NMR (151 MHz, CD_3OD) δ 172.70, 172.03, 171.62, 154.65, 151.21, 149.82, 148.27, 144.11, 136.75, 132.31, 131.40, 129.62, 129.26, 128.83, 128.63, 128.52, 128.07, 127.83, 127.47, 127.31, 125.77, 124.96, 124.48, 123.15, 122.72, 116.69, 114.96, 113.50, 108.01, 39.21, 17.18, 15.24, 0.75, 0.31.

HRMS [$C_{45}H_{43}N_4O_4Si^+$] Cal: 731.3048, Obs: 731.3025.

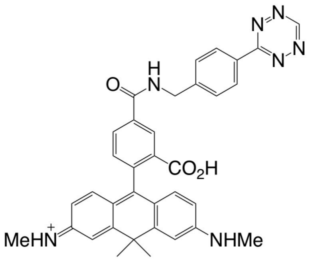

Synthesis of 580CP-Tz 3. 580CP-COOH was synthesized as previously described[5]. 580CP-COOH (5 mg, 1 eq.), TSTU (1.2 eq.) and DIPEA (2 eq.) were dissolved in DMSO and stirred at room temperature for 20 min. Tz-NH_2 (2 eq.) and DIPEA (2 eq.) were added and the stirring was continued for 2 hours until LC-MS showed completion of the reaction. The mixture was then filtered and purified by reverse phase HPLC using eluent A (H_2O with 0.1% TFA) and eluent B (CH_3CN with 0.1% TFA) to give 580CP-Tz as a purple solid (86% yield).

^{1}H NMR (600 MHz, Methanol-d_4) δ 10.31 (s, 1H), 8.79 (d, *J* = 1.8 Hz, 1H), 8.59 (d, *J* = 8.4 Hz, 2H), 8.27 (dd, *J* = 7.9, 1.9 Hz, 1H), 7.68 (d, *J* = 8.5 Hz, 2H), 7.46 (d, *J* = 7.9 Hz, 1H), 7.10 (d, *J* = 2.2 Hz, 2H), 6.96 (d, *J* = 8.8 Hz, 2H), 6.60 (dd, *J* = 9.2, 2.2 Hz, 2H), 4.78 (s, 2H), 3.03 (s, 6H), 1.81 (s, 3H), 1.70 (s, 3H).

^{13}C NMR (151 MHz, CD_3OD) δ 169.27, 168.50, 168.25, 167.66, 160.17, 160.15, 160.13, 146.36, 143.06, 137.51, 133.87, 133.32, 133.03, 132.79, 132.06, 130.33, 130.28, 122.69, 112.29, 45.35, 36.35, 32.72, 30.87.

HRMS: [$C_{35}H_{32}N_7O_3^+$] Cal: 598.2561, Obs: 598.2554.

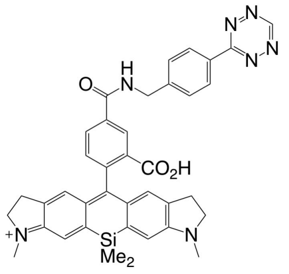

Synthesis of SiR700-Tz 4. SiR700-COOH was synthesized as previously described [6]. SiR700-COOH (5 mg, 1 eq.), TSTU (1.2 eq.) and DIPEA (2 eq.) were dissolved in DMSO and stirred at room temperature for 20 min. Tz-NH2 (2 eq.) and DIPEA (2 eq.) were added and the stirring was continued for 2 hours until LC-MS showed completion of the reaction. The mixture was then filtered and purified by reverse phase HPLC using eluent A (H_2O with 0.1% TFA) and eluent B (CH_3CN with 0.1% TFA) to give SiR700-Tz as a green solid (77% yield).

^{1}H NMR (600 MHz, Methanol-d_4) δ 10.35 (s, 1H), 8.62 (d, J = 8.2 Hz, 2H), 8.47 (s, 1H), 8.19 (d, J = 8.1 Hz, 1H), 7.69 (d, J = 8.1 Hz, 2H), 7.25 (d, J = 8.0 Hz, 2H), 6.80 (s, 2H), 6.63 (s, 2H), 4.78 (s, 2H), 3.30 – 3.24 (m, 4H), 2.91 – 2.72 (m, 10H), 0.63 (s, 3H), 0.56 (s, 3H).

^{13}C NMR (151 MHz, cd$_3$od) δ 173.31, 169.43, 168.50, 163.69, 160.13, 155.18, 146.33, 137.44, 137.00, 135.51, 135.20, 134.47, 133.28, 130.26, 130.25, 128.63, 128.62, 126.47, 126.43, 125.45, 112.58, 57.42, 45.30, 36.75, 30.12, 0.81, 0.36.

HRMS [$C_{38}H_{36}N_7O_3Si^+$] Cal: 666.2643, Obs: 666.2647.

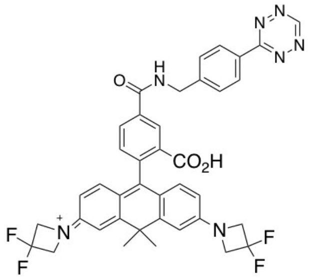

Synthesis of JF585-Tz 5. JF585-COOH[7] (Tocris Bioscience, 0.5 mg, 1 eq.), TSTU (1.2 eq.) and DIPEA (2 eq.) were dissolved in DMSO and stirred at room temperature for 20 min. Tz-NH2 (2 eq.) and DIPEA (2 eq.) were added and the stirring was continued for 2 hours until LC-MS showed completion of the reaction. The mixture was then filtered and purified by reverse phase HPLC using eluent A (H_2O with 0.1% TFA) and eluent B (CH_3CN with 0.1% TFA) to give JF585-Tz as a purple solid (87% yield).

^{1}H NMR (600 MHz, DMSO-d_6) δ 10.54 (s, 1H), 9.40 (dd, J = 5.8, 5.8 Hz, 1H), 8.40 (d, J = 8.2 Hz, 2H), 8.17 (d, J = 8.1 Hz, 1H), 8.08 (d, J = 8.1 Hz, 1H), 7.52 (d, J = 8.2 Hz, 2H), 7.47 (s, 1H), 6.83 (d, J = 2.0 Hz, 2H), 6.51 (d, J = 8.6 Hz, 2H), 6.39 (dd, J = 8.6, 2.2 Hz, 2H), 4.51 (d, J = 5.8 Hz, 2H), 4.28 (t, J = 12.3 Hz, 8H), 1.80 (s, 3H), 1.68 (s, 3H).

^{13}C NMR (151 MHz, DMSO-d_6) δ 169.49, 165.80, 165.26, 158.55, 155.62, 150.76, 146.62, 144.77, 140.60, 130.92, 128.72, 128.30, 125.51, 122.56, 121.00, 117.12, 112.74, 116.23 (t, J = 277.2 Hz), 110.42, 63.32 (t, J = 24.9 Hz), 44.55, 38.38, 34.78, 33.52.

HRMS [$C_{39}H_{32}F_4N_7O_3^+$] Cal: 722.2497, Obs: 722.2496.

Compound **6** was synthesized according to previous reports[7]:

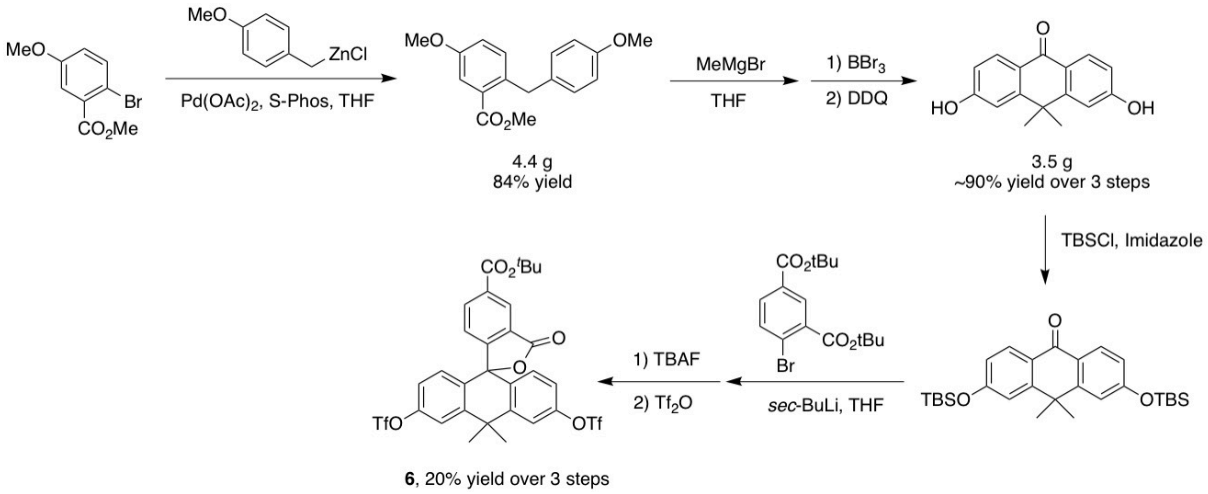

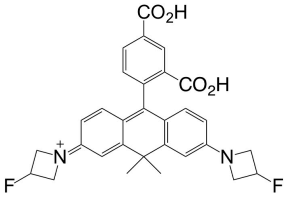

Synthesis of Yale595-COOH 7. Yale595-COOH **7** was prepared from bis-triflate **6** as described previously[7]. To a Schlenk tube was added Pd_2dba_3 (0.1 eq.), X-phos (0.3 eq.), compound **6** (1 eq., 130 mg), 3-fluoroazetidine hydrochloride (2.2 eq.), Cs_2CO_3 (5 eq.), and 4 mL dioxane. The mixture was stirred under N_2 at 100 ^oC for 5 h. The mixture was filtered through a pad of Celite, concentrated and purified by silica gel to obtain the product as a dark-purple solid (86 % yield).

^1H NMR (600 MHz, Methanol-d_4) δ 8.85 (s, 1H), 8.37 (d, J = 7.9 Hz, 1H), 7.42 (d, J = 7.9 Hz, 1H), 7.01 – 6.92 (m, 4H), 6.46 (d, J = 9.1 Hz, 2H), 5.63 – 5.59 (m, 1H), 5.54 – 5.50 (m, 1H), 4.66 – 4.56 (m, 4H), 4.38 (dd, J = 23.4, 11.6 Hz, 4H), 1.88 (s, 3H), 1.76 (s, 3H).

^{13}C NMR (151 MHz, CD_3OD) δ 168.83, 168.49, 158.32, 157.28, 157.27, 137.94, 135.26, 134.38, 133.56, 133.17, 132.16, 122.90, 113.47, 111.35, 84.39 (d, J = 203.7 Hz), 61.34 (d, J = 26.3 Hz), 43.49, 36.30, 32.81.

HRMS [$C_{30}H_{27}F_2N_2O_4^+$] Cal: 517.1933, Obs: 517.1934.

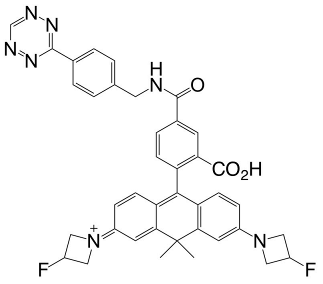

Synthesis of Yale595-Tz 8. Yale595-COOH (5 mg, 1 eq.), TSTU (1.2 eq.) and DIPEA (2 eq.) were dissolved in DMSO and stirred at room temperature for 20 min. Tz-NH_2 (2 eq.) and DIPEA (2 eq.) was added and the stirring was continued for 2 hours until LC-MS showed no remaining starting material. The mixture was then filtered and purified by reverse phase HPLC using eluent A (H_2O with 0.1% TFA) and eluent B (CH_3CN with 0.1% TFA) to give Yale595-Tz as a purple solid (91% yield).

^{1}H NMR (600 MHz, DMSO-d_6) δ 10.59 (s, 1H), 9.52 (t, J = 6.0 Hz, 1H), 8.54 (s, 1H), 8.50 (d, J = 8.3 Hz, 2H), 8.23 (dd, J = 8.1, 1.5 Hz, 1H), 7.66 (d, J = 8.4 Hz, 2H), 7.17 (d, J = 7.9 Hz, 1H), 6.74 (d, J = 2.3 Hz, 2H), 6.49 (d, J = 8.6 Hz, 2H), 6.33 (dd, J = 8.6, 2.4 Hz, 2H), 5.57 – 5.51 (m, 1H), 5.47 – 5.41 (m, 1H), 4.67 (d, J = 5.9 Hz, 2H), 4.19 (ddd, J = 20.8, 9.4, 5.8 Hz, 4H), 3.93 (ddd, J = 24.2, 9.3, 2.4 Hz, 4H), 1.80 (s, 3H), 1.69 (s, 3H).

^{13}C NMR (151 MHz, DMSO-d_6) δ 169.28, 165.42, 164.89, 158.11, 157.66, 151.41, 151.40, 146.06, 144.58, 135.29, 134.62, 130.48, 128.24, 128.22, 127.86, 126.03, 123.90, 123.42, 119.46, 111.32, 108.87, 83.46 (d, J = 199.9 Hz), 59.28 (d, J = 23.3 Hz), 48.59, 42.71, 37.86, 34.38, 33.11.

HRMS: [$C_{39}H_{34}F_2N_7O_3^+$] Cal: 686.2686, Obs: 686.2683.

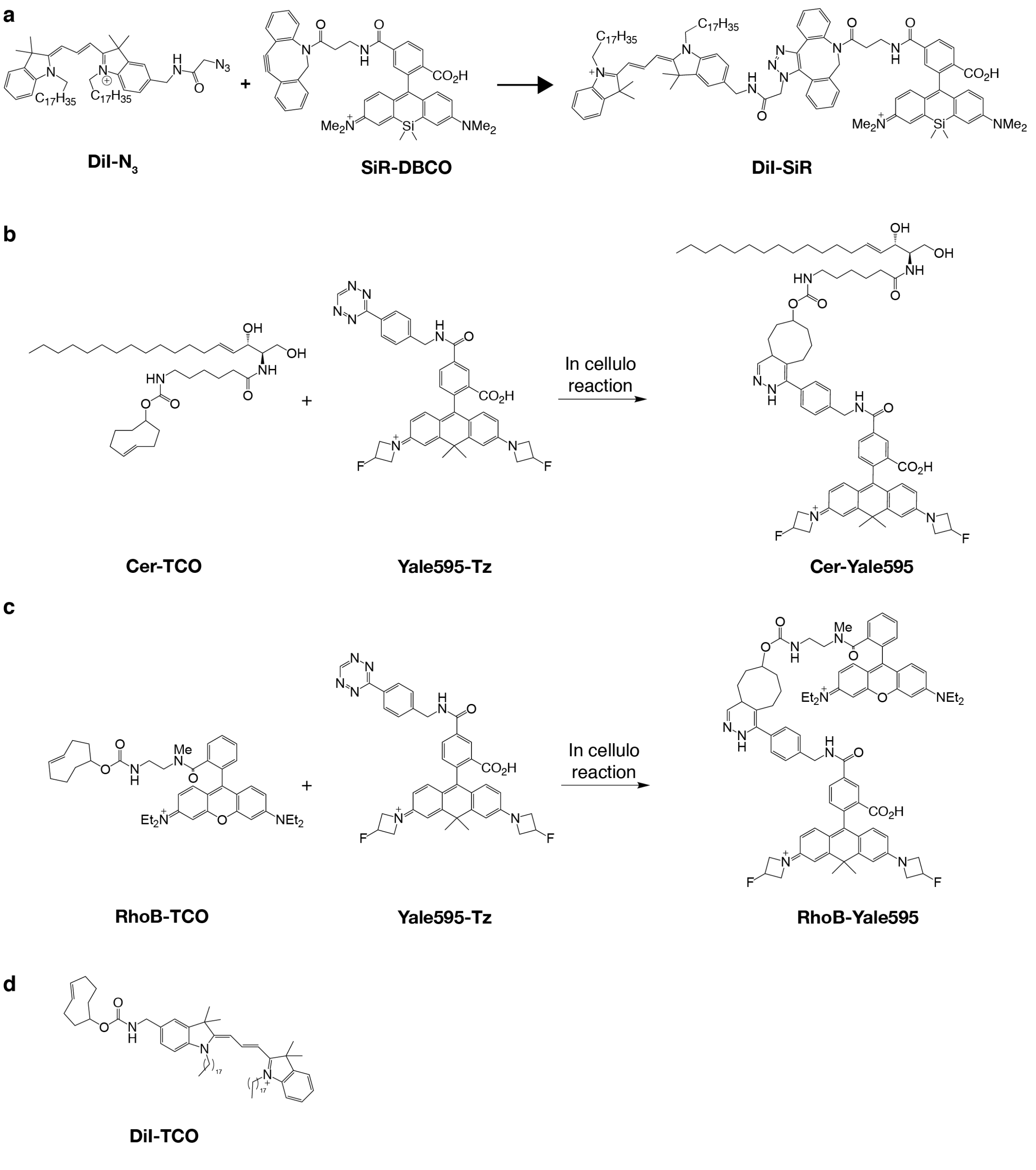

Supplementary Figure 1. Chemical structures and reactions used for labeling of organelle membranes. (a) In cellulo strain-promoted alkyne-azide cycloadditions between DiI-N_3 and SiR-DBCO yields DiI-SiR. (b) In cellulo inverse electron-demand Diels-Alder reaction between Cer-TCO and Yale595-Tz yields Cer-Yale595. (c) In cellulo inverse electron-demand Diels-Alder reaction between RhoB-TCO and Yale595-Tz yields RhoB-Yale595. (d) Structure of DiI-TCO.

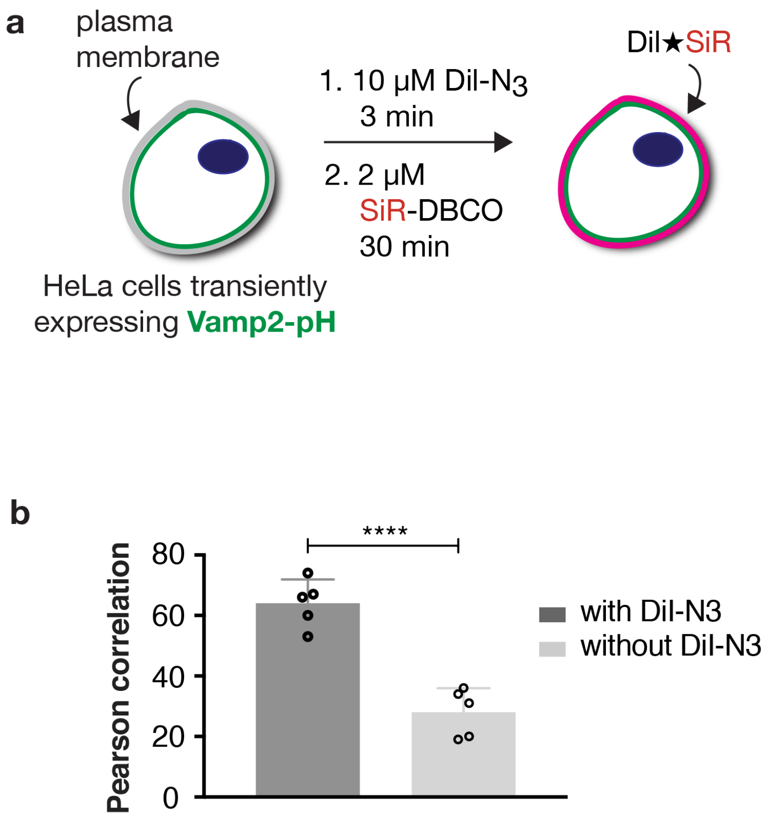

Supplementary Figure 2. Labeling of plasma membrane with DiI-N_3 and SiR-DBCO. (a) Schematic illustration of the the in vivo labeling protocol. (b) Pearson correlation between Vamp2-pH and DiI-SiR (dark gray) or Vamp2-pH and SiR-DBCO (light grey). (mean±standard deviation, N = 5), ****P≤ 0.0001, unpaired t-test, two-tailed.

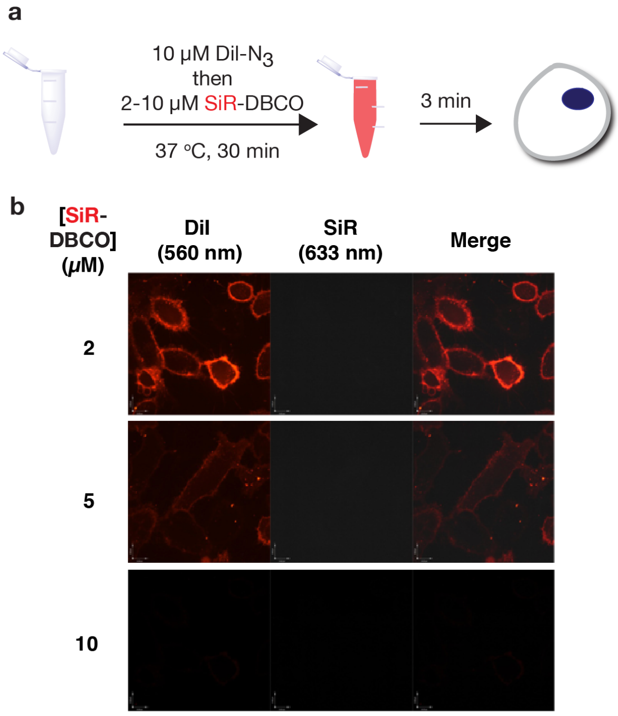

Supplementary Figure 3. In vitro pre-mixing of DiI-N_3 and SiR-DBCO and labeling of plasma membrane. (a) Schematic illustration of the in vitro mixing protocol. 10 μM DiI-N_3 in PBS with 1% casein was mixed with different concentrations of SiR-DBCO in PBS with 1% casein in an eppendorf tube and incubated at 37 °C for 30 min. The mixture was then added to HeLa cells and incubated at 37 °C for 3 min and imaged under a spinning disk confocal microscope. The formation of product DiI-SiR in vitro was confirmed by LC-MS. (b) Images of 10 μM DiI-N_3 pre-mixed with different concentrations of SiR-DBCO. No plasma membrane labeling was detected in the SiR (633 nm) channel, indicating the pre-mixed product does not localize on the plasma membrane.

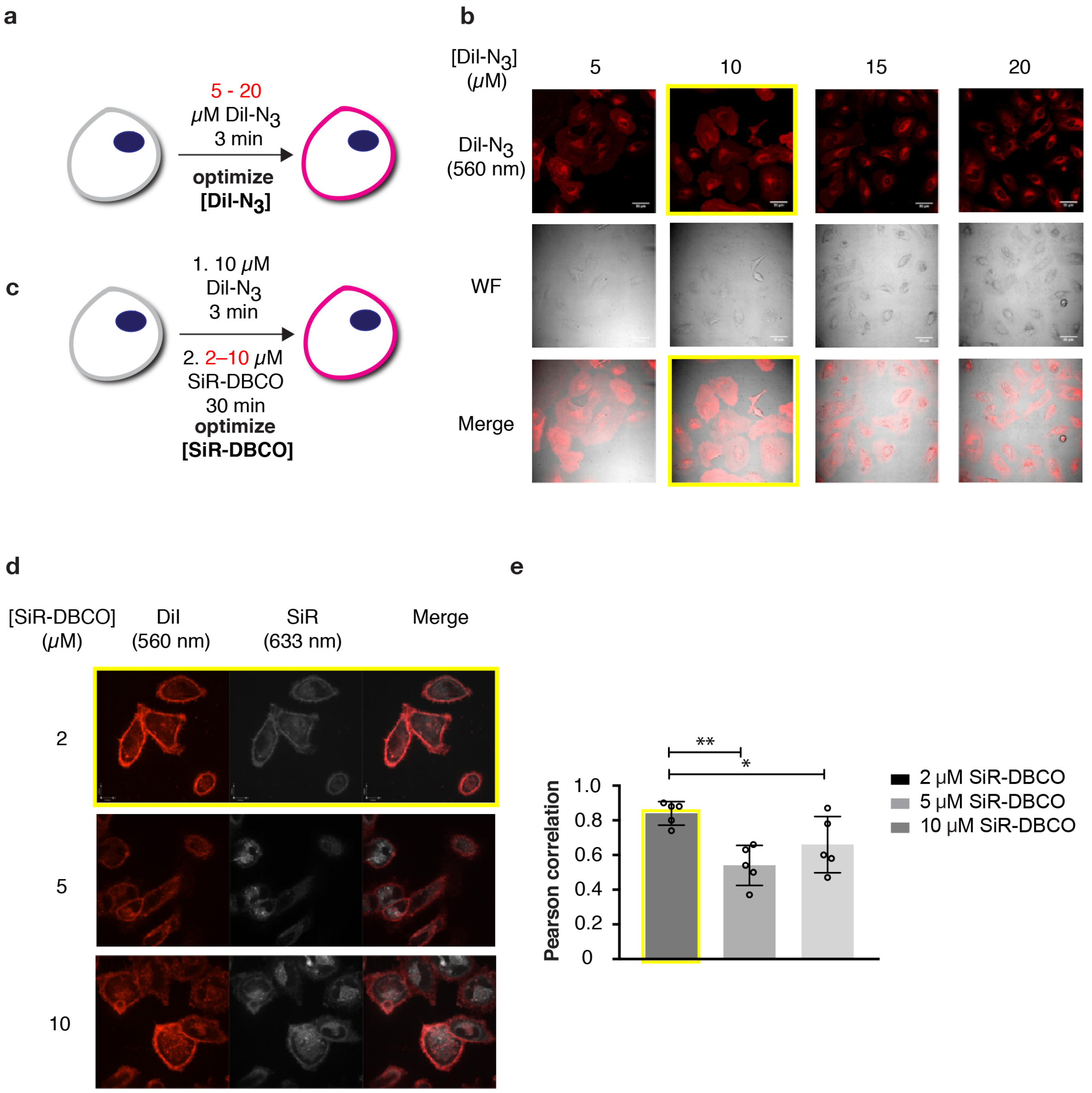

Supplementary Figure 4. Optimization of labeling conditions with DiI-N_3 and SiR-DBCO. (a) Schematic illustration of HeLa cells labeled with different concentrations of DiI-N_3 (b) Images of HeLa cells labeled with X μM DiI-N_3. When 5 μM DiI-N_3 was used, not all cells are universily labeled. When 15 or 20 μM DiI-N_3 was used, nonspecific labeling in the cytosol was observed. (c) Schematic illustration of HeLa cells labeled with 10 μM DiI-N_3 and different concentrations of SiR-DBCO. (d) Confocal images of HeLa cells labeled with 10 μM DiI-N_3 and different concentrations of SiR-DBCO. Images of both DiI (560 nm) channel and SiR (633 nm) channel was recorded. (e) Pearson correlation between DiI channel and SiR channel. (mean±standard deviation, N = 5), *P = 0.0328, **P = 0.0011, unpaired t-test, two-tailed.

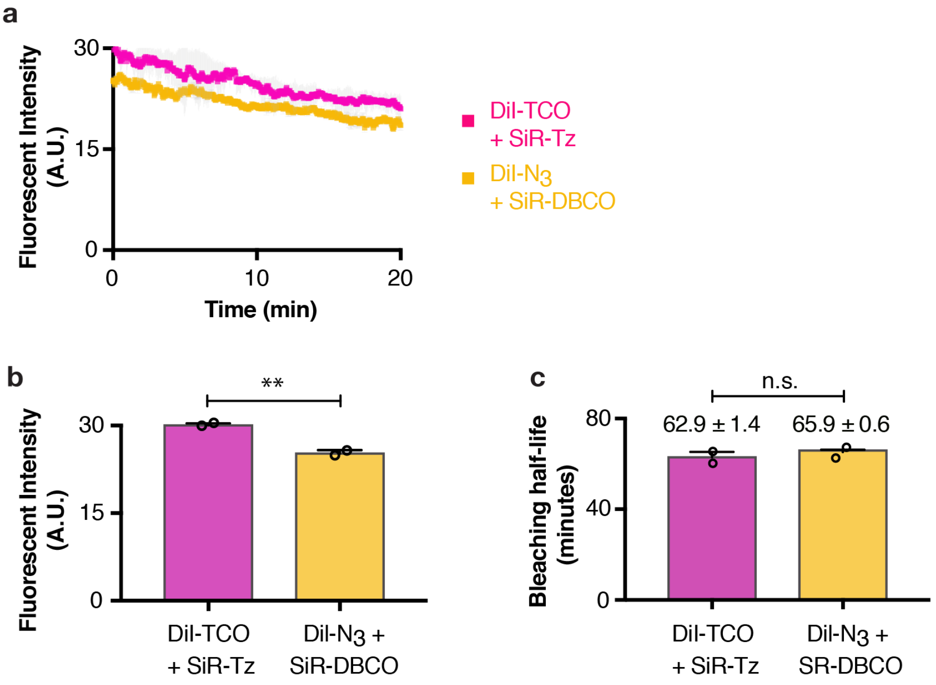

Supplementary Figure 5. Comparison of TCO-tetrazine ligation and Cu-free SPAAC click chemistry.
(a) Plot illustrating fluorescence intensity of HeLa cells labled with DiI-TCO+SiR-Tz or DiI-N_3+SiR-DBCO over time (mean±standard deviation, n = 2 region of interest, N = 1 cell). (b) Fluoresecent intensity of a at time 0. **P = 0.0096, unpaired t-test, two-tailed (c) Bleaching half-life calculated from a single exponential fit to the photobleaching curves in a (mean ± standard deviation., n = 2 region of interest, N = 1 cell). n.s. = not significant.

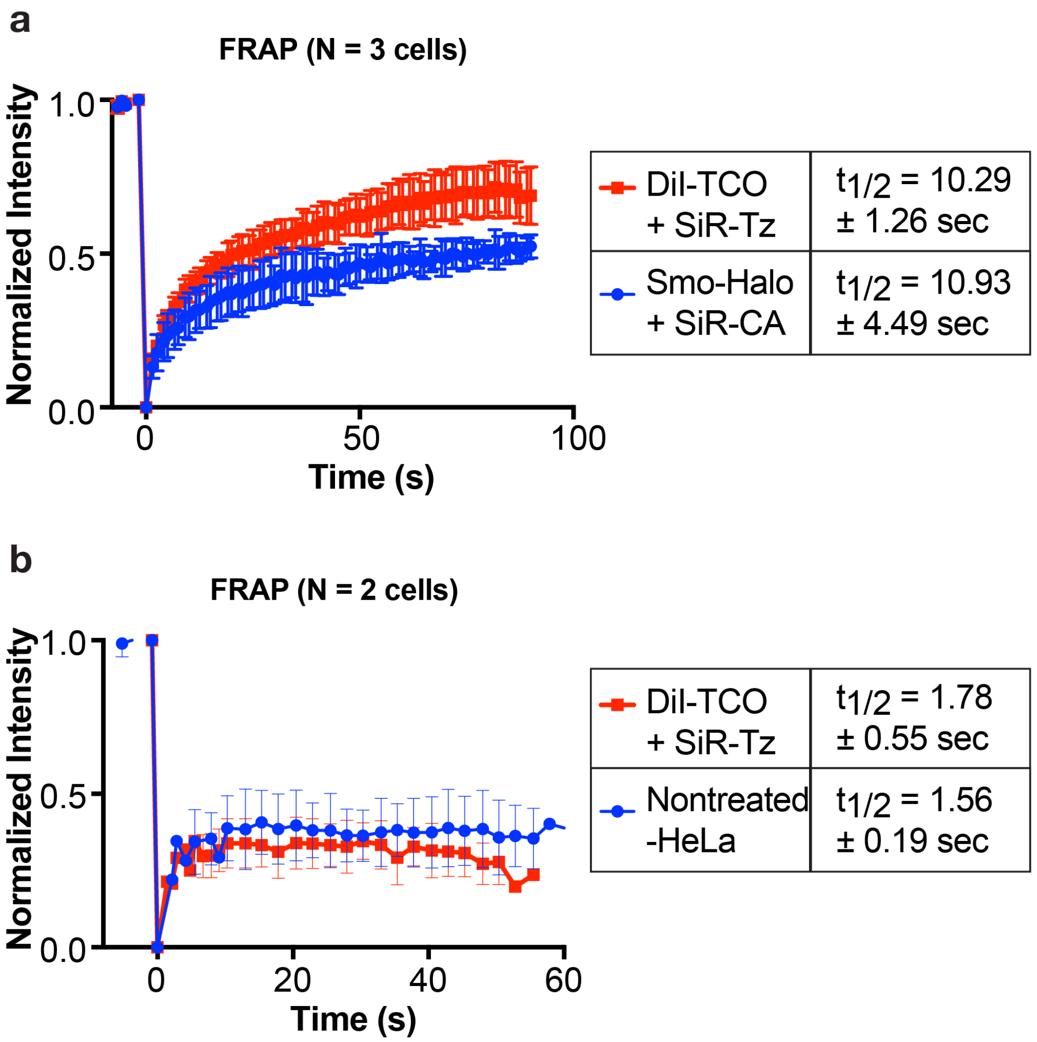

Supplementary Figure 6. Fluorescence recovery after photobleaching (FRAP) experiments. (a) Plot illustrating normalized fluorescence intensity of RPE cells labled with Smo-Halo+SiR-CA or DiI-TCO+SiR-Tz over time (mean± standard deviation (SD), N = 3 cells). RPE cells stably expressing Smo-Halo were labeled with either 2 μM SiR-CA or 10 μM DiI-TCO + 2 μM SiR-Tz and performed the FRAP experiment. Photobleaching was performed with the built-in FRAP unit. A region of interest was photobleached using 40 scans of the 650 nm laser (SiR channel) at 100% laser power. Images of a single XY-plane were taken at 0.8 Hz with an exposure time of 167 ms. Quantification was performed with Velocity. The resulting curves were fit to an exponential growth equation using Velocity Analysis. (b) Plot illustrating normalized fluorescence intensity of Laurdan (405 nm) of non-treated HeLa cells or HeLa cells labled with DiI-TCO+SiR-Tz over time (mean±SD, N = 2 cells). HeLa cells labeled with either 5 μM Laurdan or 10 uM DiI-TCO + 2 uM SiR-Tz + 5 μM Laurdan. Photobleaching was performed with the built-in FRAP unit. A region of interest was photobleached using 40 scans of the 405 nm laser (Laurdan channel) at 100% laser power. Images of a single XY-plane were taken at 0.5 Hz with an exposure time of 210 ms. Quantification was performed with Velocity.

a

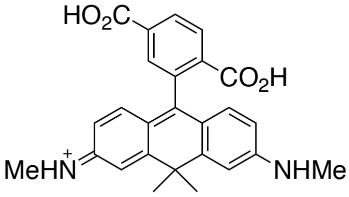

580CP

Angew. Chem. Int. Ed. 55, 3290 (**2016**)

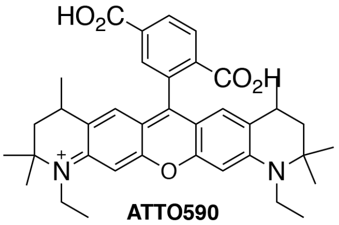

ATTO590

PMC Biophys. 3, 4 (**2010**); *Nat. Commun.* 7 (**2016**) DOI: 10.1038/ncomms10778

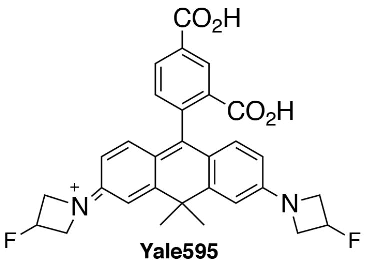

Yale595

This work

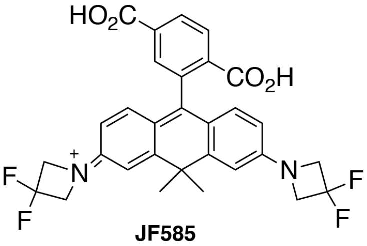

JF585

Nat. Methods 14, 987 (**2017**)

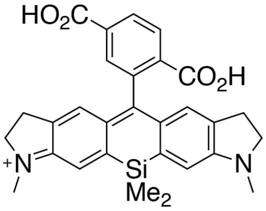

SiR700

J. Am. Chem. Soc. 138, 9365 (**2016**)

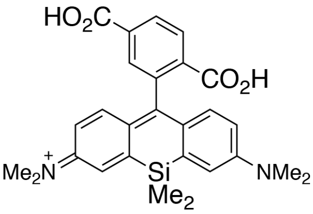

SiR

Nat. Chem. 5, 132 (**2013**)

b

Dye	Ex (nm)	Em (nm)	ε_{max} ($M^{-1}cm^{-1}$)	QY	Relative brightness
580CP	582	607	90000	0.69	1.55
ATTO590	593	622	120000	0.80	2.4
JF585	585	609	156000	0.78	3.05
SiR700	687	716	100000	0.13	0.33
Yale595	597	621	88000	0.76	1.67
SiR	645	677	100000	0.4	1

c

HIDE probe	Ex (nm)	Em (nm)
DiI-N_3	549	565
RhoB-TCO	540	568

Supplementary Figure 7. Chemical structures of STED dyes used in this work. (a) Chemical structures and references. (b) Photophysical properties, Ex: excitation wavelength; Em: emission wavelength; ε_{max}: extinction coefficient; QY: quantum yield. (c) Photophysical proerties.

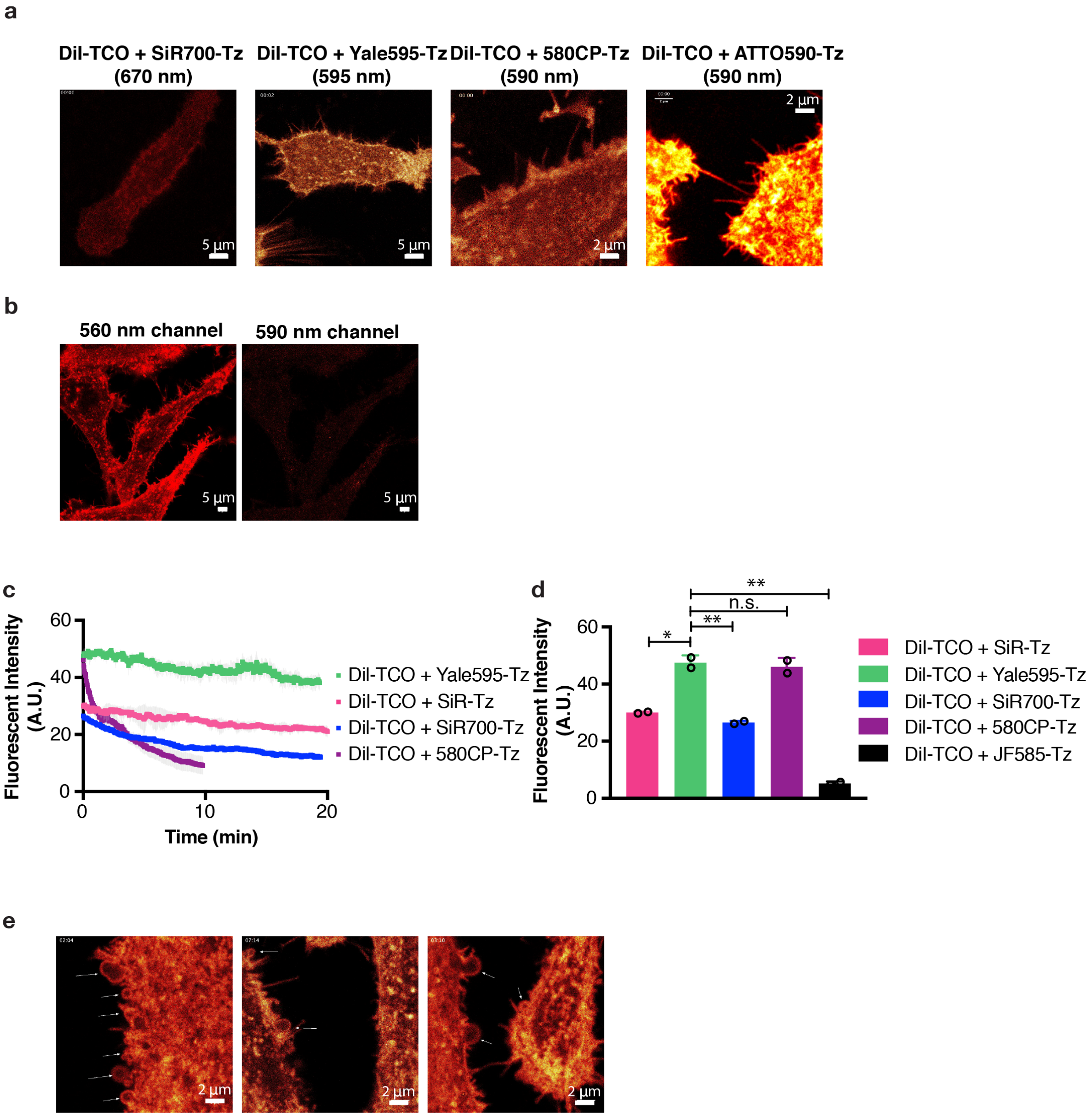

Supplementary Figure 8. Live-cell nanoscopy with Yale595, SiR700, CP580, Atto590, or JF585 (a) Representative images of plasma membrane labeled with DiI-TCO and Yale595-Tz, SiR700-Tz, 580CP-Tz, or Atto590-Tz. (b) Representative images of plasma membrane labeled with DiI-TCO and JF585-Tz. Image of DiI (560 nm) channel showed DiI-JF585 localized on the plasma membrane. Image of JF585 channel (590 nm) showed weak fluorescence. (c) Plot illustrating fluorescence intensity of 580CP (purple), SiR (red), SiR700 (blue) and Yale595 (green) over time (mean± standard deviation (SD), n = 2 region of interest (ROI), N = 1 cell). (d) Fluoresecent intensity of d at time 0 (mean±SD, n = 2 ROI, N = 1 cell). *P = 0.0108, **P (580CP) = 0.0078, **P (JF585) = 0.0020, n.s. = not significant, unpaired t-test, two-tailed (e) Cell membrane blebbing when exiting Att0590-Tz. Cells were labeled with Atto590Tz followed by DiI-TCO and imaged by STED confocal microscopy (excitation at 595). As shown by the white arrows (three different cells) there was high toxicity as assessed by membrane blebs when using this dye.

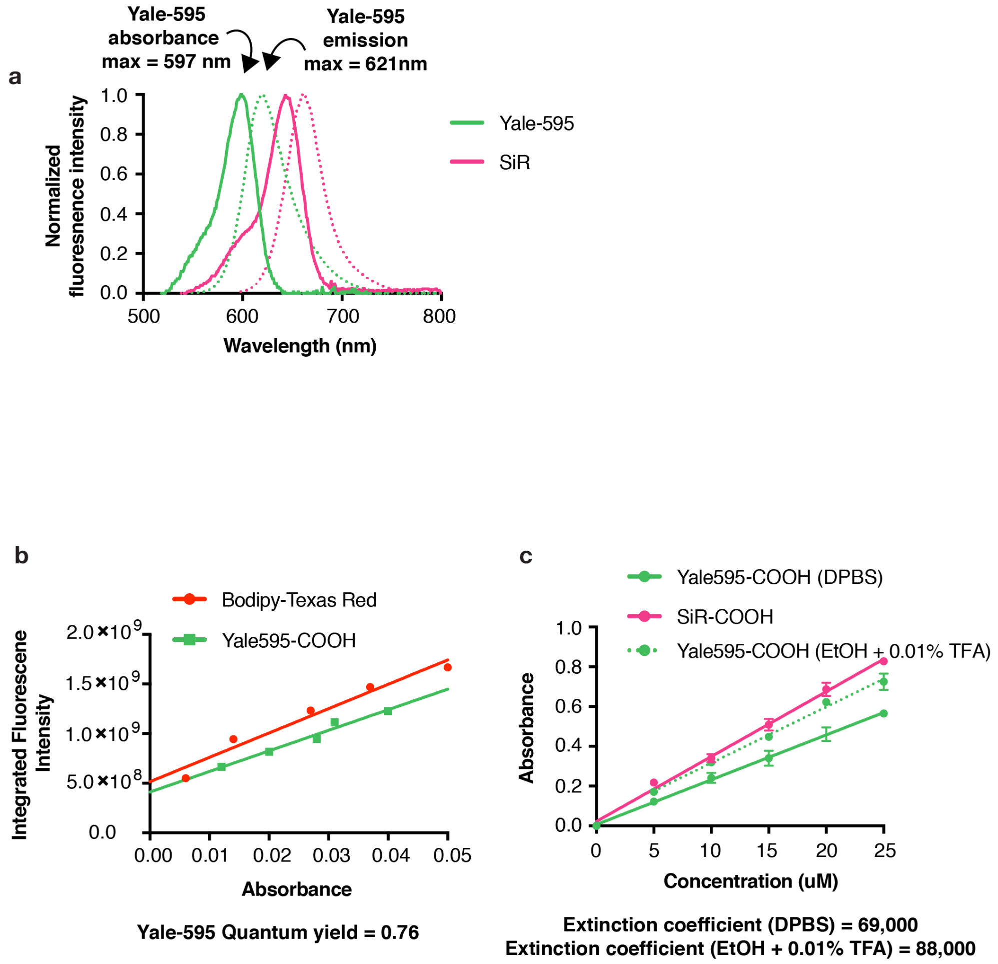

Supplementary Figure 9. Photophysical properties of Yale-595. (a) Fluorescence excitation (solid lines) and emission (dashed lines) spectra of Yale-595 (green) and SiR (pink). (b) Integrated fluorescence of Yale-595 (green) and Bodipy-Texas Red (red) as a function of absorbance at 550 nm. (c) Standard curve for Yale-595 (green) and SiR (pink) in DPBS (solid) or EtOH with 0.1% trifluoroacetic acid (dash) (mean ± standard deviation, N = 3).

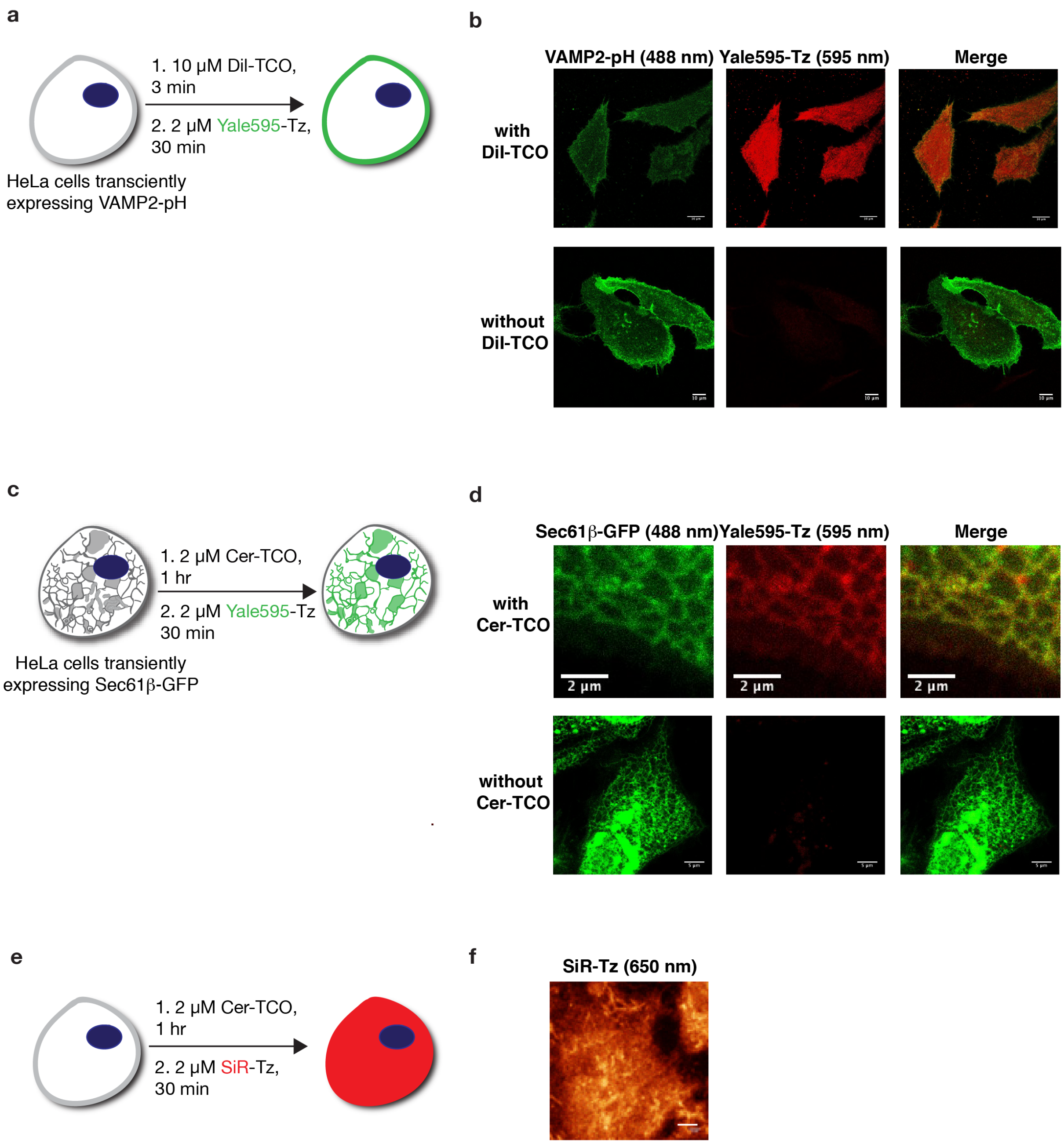

Supplementary Figure 10. Colocalization of organelles labeled with HIDE probes containing Yale595 with protein marker. (a) Schematic illustration of HeLa cells transiently expressing VAMP2-pH labeled with Dil-TCO and Yale595-Tz. (b) Representative images of plasma membrane labeled with VAMP2-pH (488 nm channel) and Yale595 (595 nm channel). Scale bars: 10 µm.(c) Schematic illustration of HeLa cells transciently expressing Sec61β-GFP labeled with Cer-TCO and Yale595-Tz. (d) Representative images of ER labeled with Sec61β-GFP (488 nm channel) and Yale595 (595 nm channel). (e) Schematic illustration of HeLa cells labeled with Cer-TCO and SiR-Tz. (f) Representative images of HeLa cells labeled with Cer-TCO and SiR-Tz (650 nm). Scale bar: 5 µm

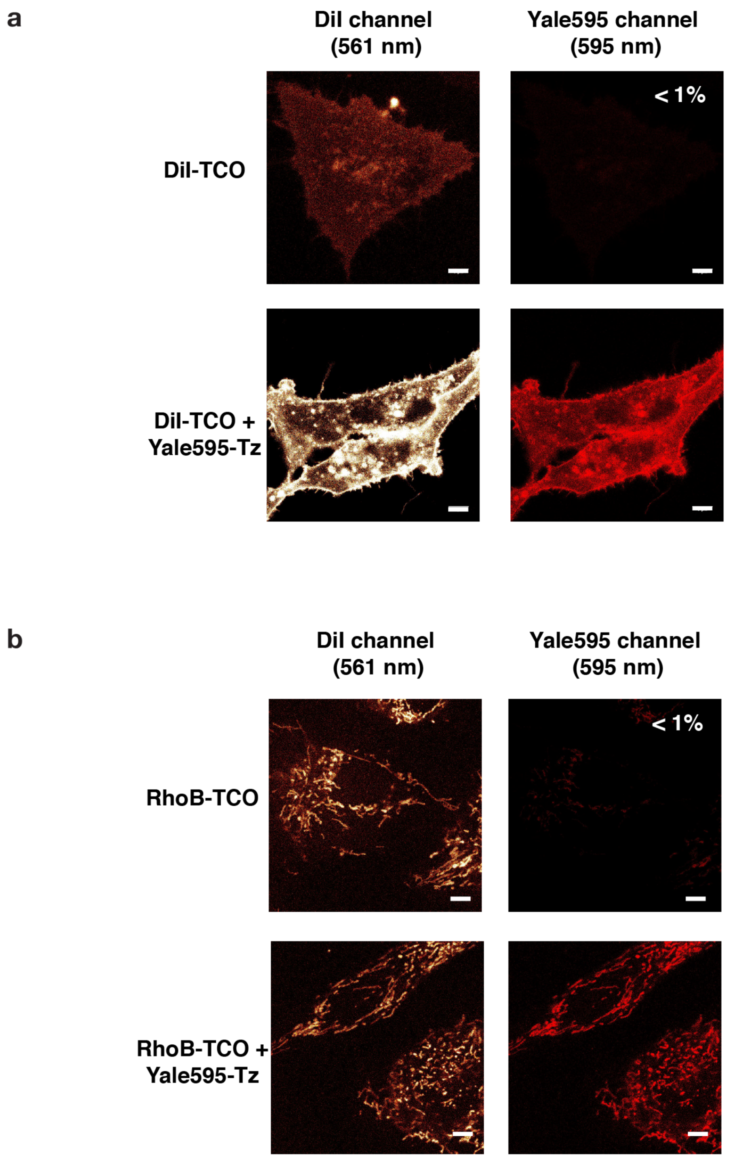

Supplementary Figure 11. Measurement of crosstalk between 561 nm and 595 nm channels. (a) Selected example illustrating mininal crosstalk of DiI-TCO in 595 nm channel. HeLa cells were labeled with either DiI-TCO alone (10 μM) or with DiI-TCO + Yale595-Tz (10 μM + 2 μM), and imaged in both the 561 nm channel (to detect DiI) and the 595 nm channel (to presumably detect only Yale-595) using the same STED setting described in the manuscript (10% 595 nm excitation, 30% 775 nm depletion, detection window: 605-625 nm). Scale bars: 10 µm.(b) Selected example illustrating mininal crosstalk of RhoB-TCO in 595 nm channel. HeLa cells were labeled with either RhoB-TCO alone (10 μM) or with RhoB-TCO + Yale595-Tz (10 μM + 2 μM), and imaged in both the 561 nm channel (to detect DiI) and the 595 nm channel (to presumably detect only Yale-595) using the same STED setting described in the manuscript (10% 595 nm excitation, 30% 775 nm depletion, detection window: 605-625 nm). Scale bars: 10 µm.

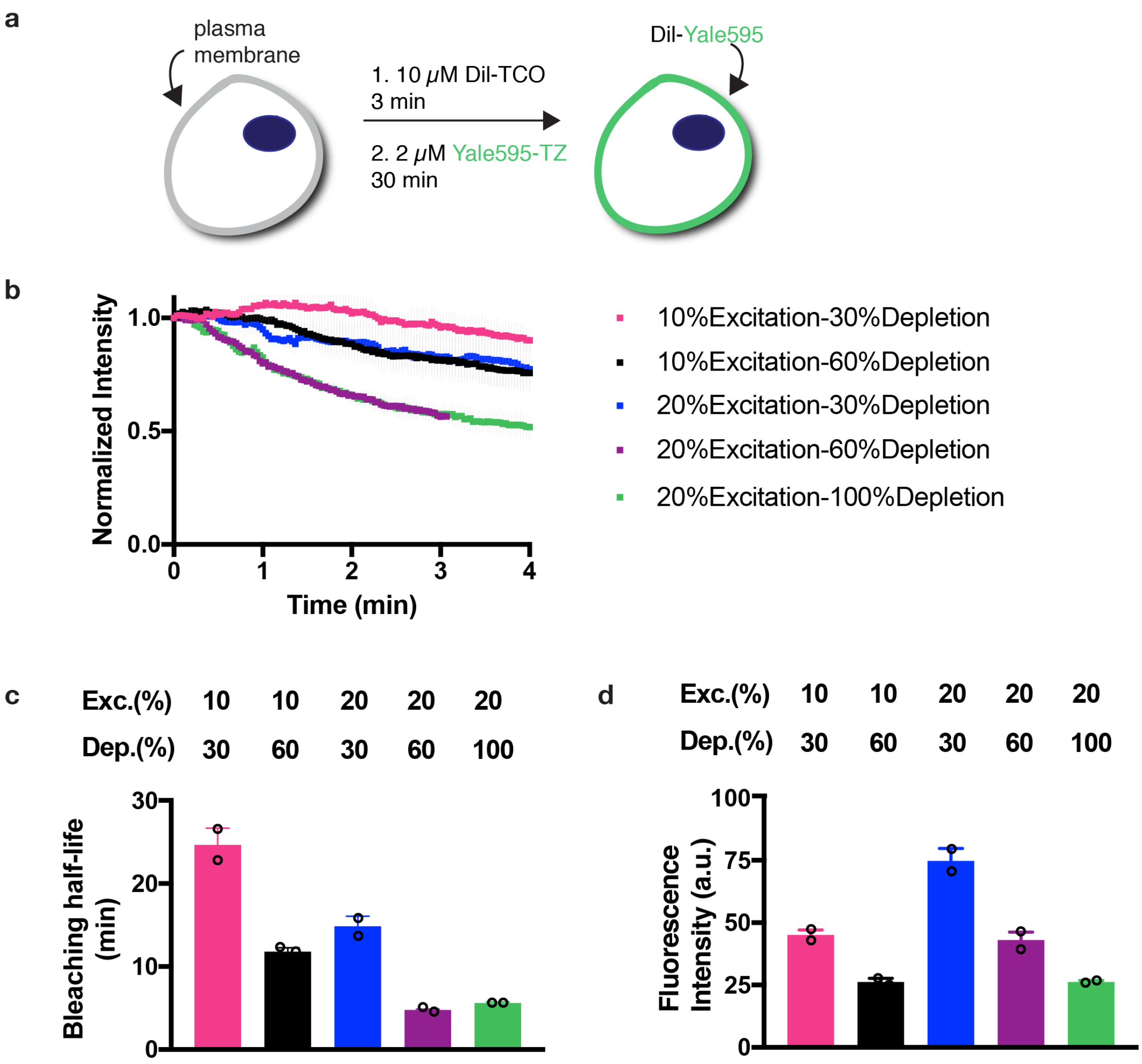

Supplementary Figure 12. Photobleaching curve of Yale595 under different imaging conditions. (a) Schematic illustration of HeLa cells labeled with DiI-TCO+Yale595-Tz. (b) Plot illustrating normalized fluorescence intensity of HeLa cells over time under different imaging conditions. (c) Bleaching half-life calculated from a single exponential fit to the photobleaching curves in b (mean±standard deviation, N = 2 cells), (d) Initial fluorecence intensity of HeLa cells under different imaging conditions (mean±standard deviation, N = 2 cells).

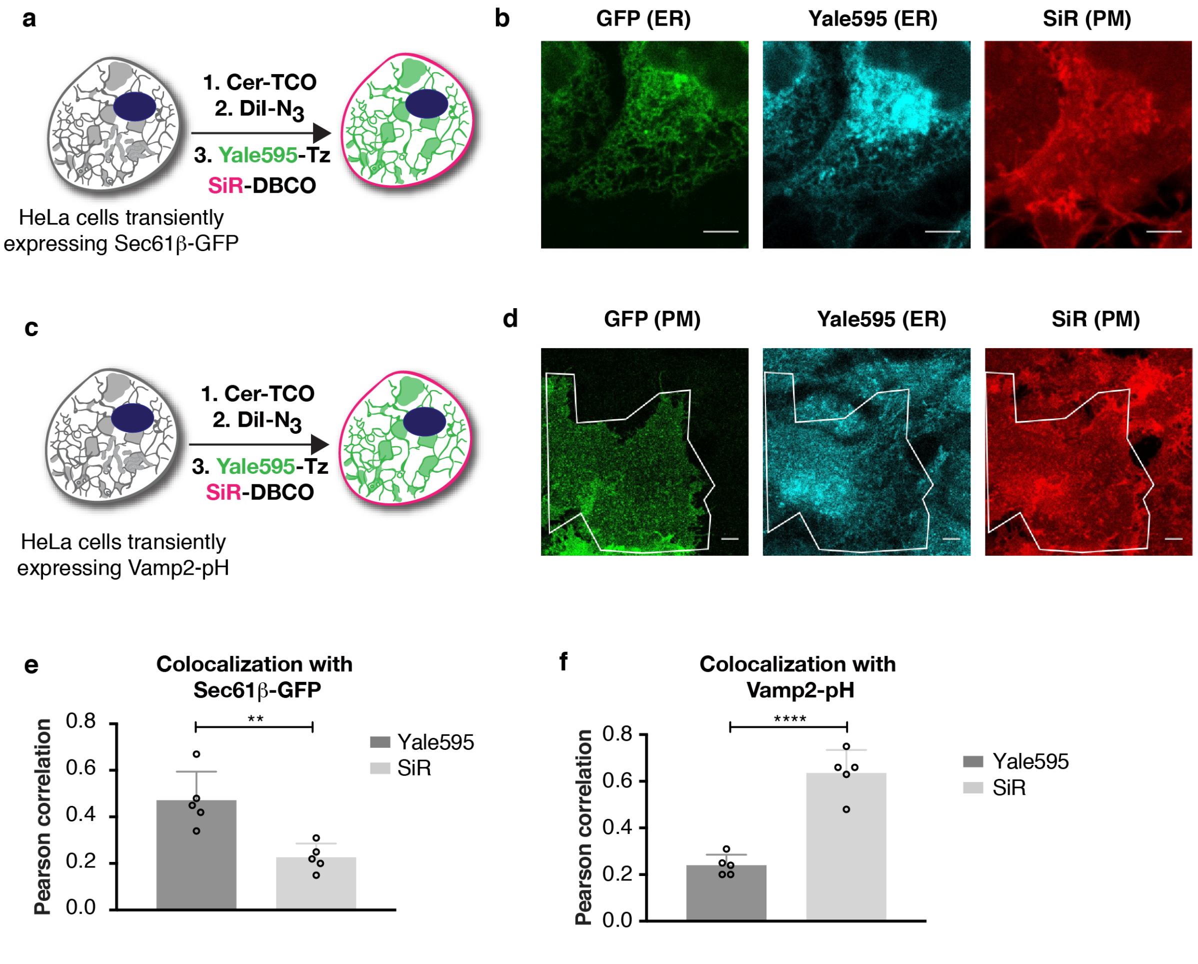

Supplementary Figure 13. Colocalization of two-color HIDE probe labeling of PM and ER with organelle marker
(a) Schematic illustration of HIDE probe PM and ER labeling of HeLa cells transiently expressing Sec61b-GFP. (b) Representative images of PM and ER labeling in three channels. Scale bars: 2 µm.(c) Schematic illustration of HIDE probe PM and ER labeling of HeLa cells transciently expressing Vamp2-pH. (d) Representative images of PM and ER labeling in three channels. Scale bars: 2 µm. (e) Cer-Yale595 still colocalize with Sec61b-GFP (ER marker) with two-color HIDE labeling protocol. (mean±standard deviation (SD), N = 5), **P = 0.0076. (f) DiI-SiR still colocalize with Vamp2-pH (PM marker) with two-color HIDE labeling protocol. (mean±SD, N = 5), ****P ≤ 0.0001, unpaired t-test, two-tailed.

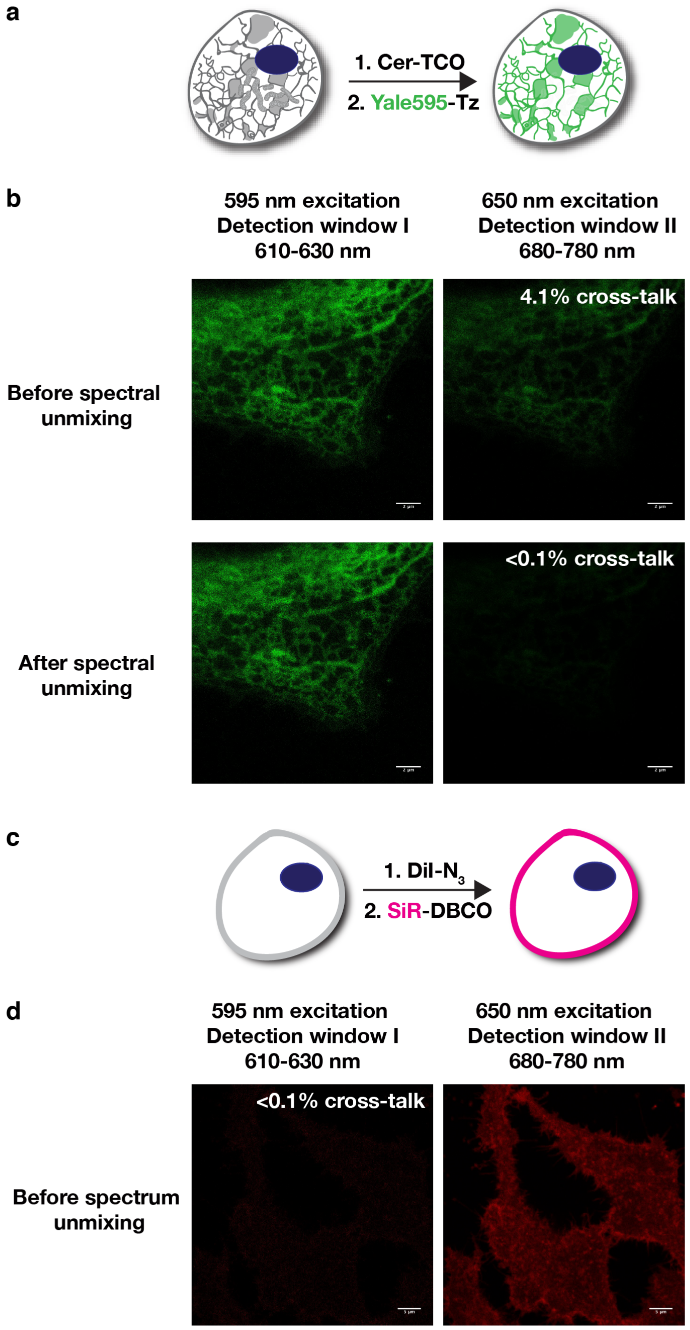

Supplementary Figure 14. Measurement of cross-talk between two channels. (a) Schematic illustration of ER labeled with Cer-TCO and Yale595-Tz. (b) STED Images of ER labeled with Cer-Yale595. Images in both 590 nm and 650 nm channel was recorded and cross-talk percentages was calculated as the ratio of fluorescent intensity between two channels. Spectral unmixing was done using a imagej plugin SpectrumUnmixing. Scale bars: 2 µm. (c) Schematic illustration of plasma membrane labeled with DiI-N_3 and SiR-DBCO. (d) STED Images of ER labeled with DiI-SiR. Images in both 590 nm and 650 nm channel was recorded. Scale bars: 5 µm.

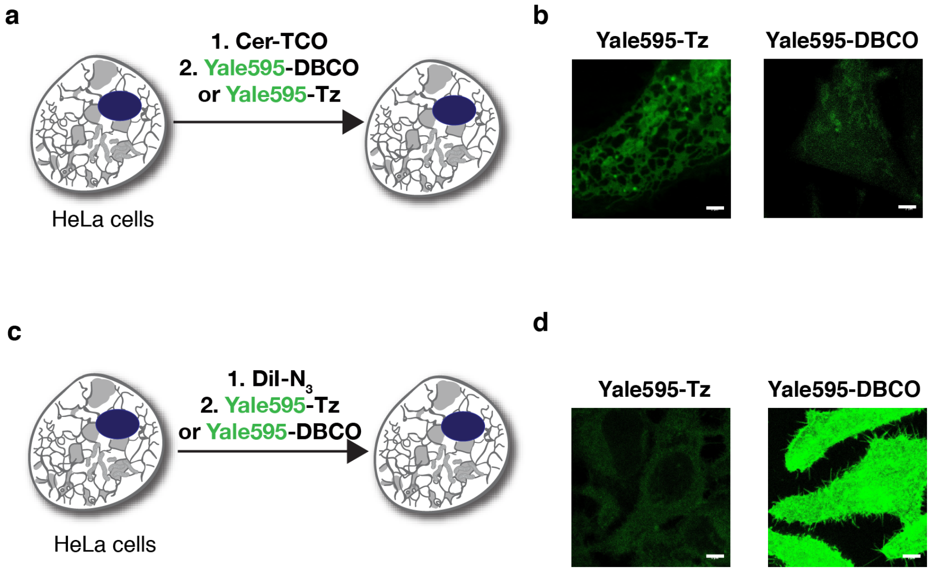

Supplementary Figure 15. Study of cross reactivity of different probes. (a) Schematic illustration of HeLa cells treated with Cer-TCO followed by Yale595-DBCO or Yale595-Tz. (b) Representative images of Yale595 channel (595 nm). Scale bars: 2 µm.(c) Schematic illustration of HeLa cells treated with DiI-N_3 followed by Yale595-Tz or Yale595-DBCO. (d) Representative images of Yale595 channel (595 nm). Scale bars: 5 µm.

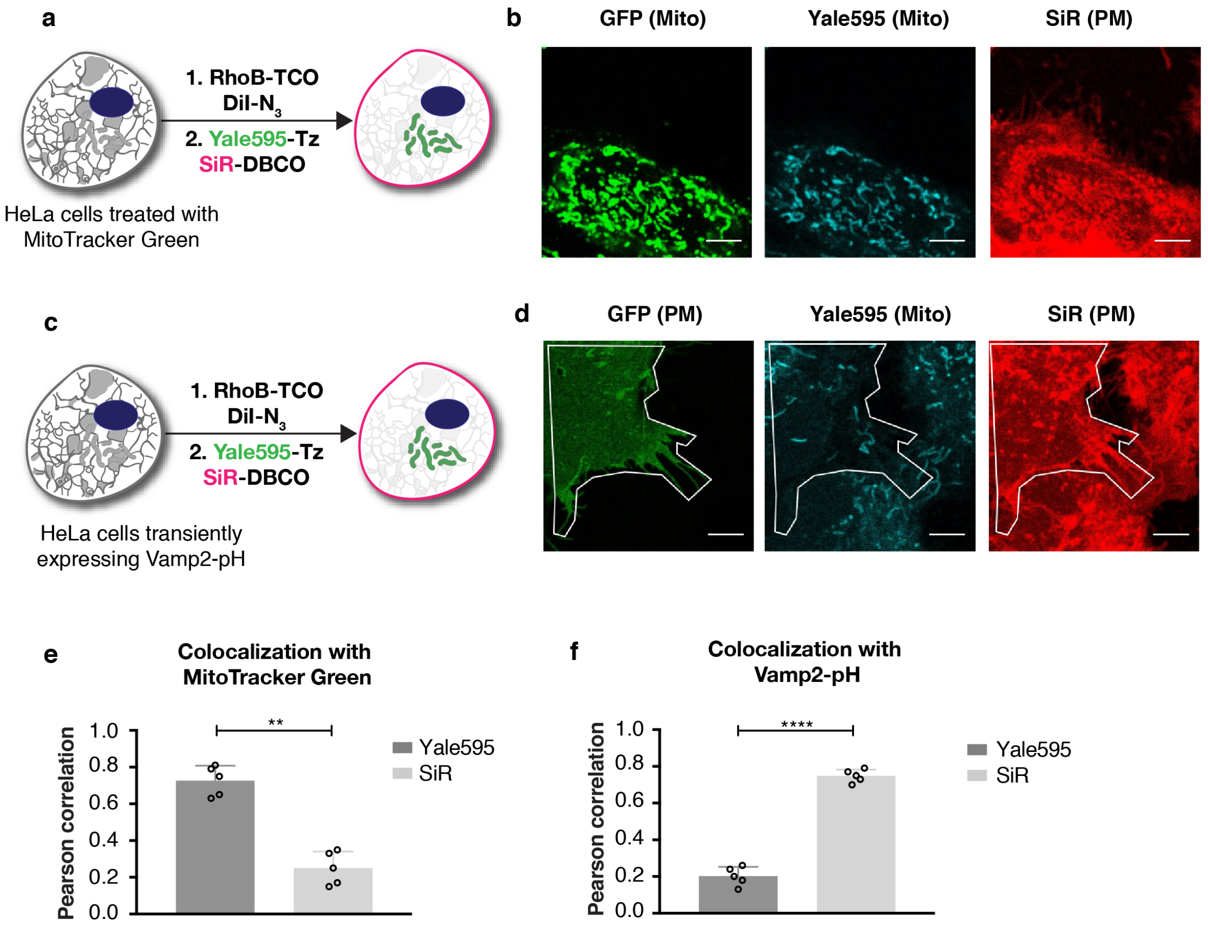

Supplementary Figure 16. Colocalization of two-color HIDE probe labeling of PM and Mitochondria with organelle marker. (a) Schematic illustration of HIDE probe PM and mitochondria labeling of HeLa cells treated with MitoTracker Green. (b) Representative images of PM and Mitochondria labeling in three channels. (c) Schematic illustration of HIDE probe PM and Mitochondria labeling of HeLa cells transiently expressing Vamp2-pH. Scale bars: 2 μm. (d) Representative images of PM and Mitochondria labeling in three channels. Scale bars: 2 μm.(e) RhoB-Yale595 still colocalize with MitoTracker Green with two-color HIDE labeling protocol. (mean±standard deviation, N = 5), **P = 0.0015, unpaired t-test, two-tailed. (f) DiI-SiR still colocalize with Vamp2-pH (PM marker) with two-color HIDE labeling protocol. (mean±standard deviation, N = 5), ****P ≤ 0.0001, unpaired t-test, two-tailed.

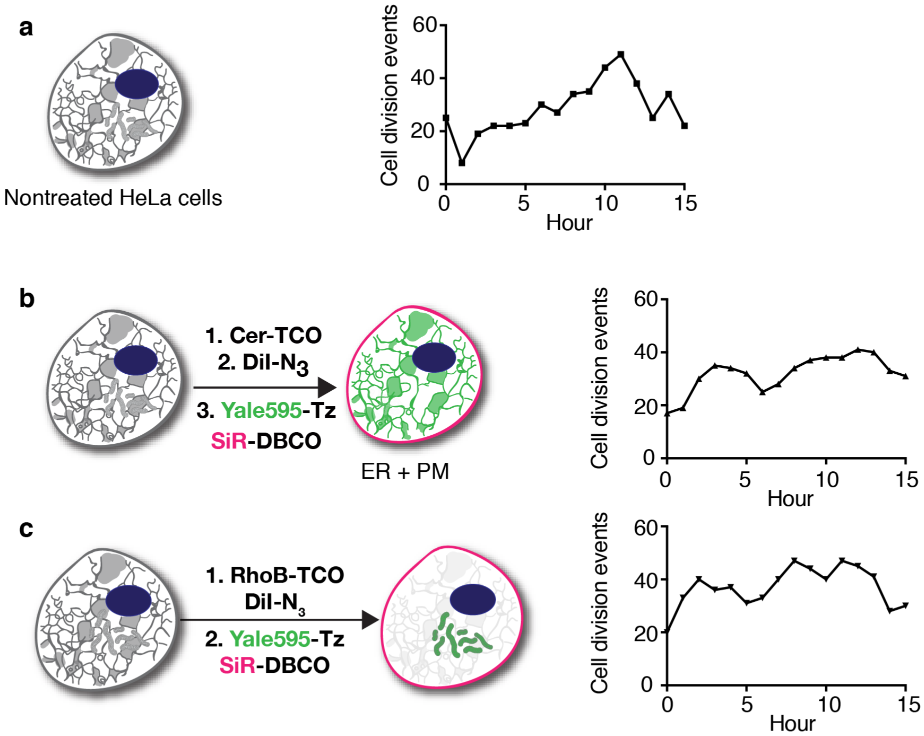

Supplementary Figure 17. Cell toxicity study of two-color HIDE probes. (a) Number of cell division events of nontreated HeLa cells. The cells were monitored by an Invitrogen™ EVOS™ FL Auto 2 Imaging system equipped with temperature, humidity, and atmosphere controls for live cell imaging. The sample was kept at 37 °C with 5% CO2 and imaged using a 20x objective and a Highsensitivity 1.3 MP CMOS monochrome camera (1,328 x 1,048 pixels) every 10 minutes for 15 h using bright field. Each timepoint indicates the number of cell division events in that hour. (b) Number of cell division events of HeLa cells treated with Cer-TCO and DiI-N_3 then Yale595-Tz and SiR-DBCO. Cells were washed and incubated in DMEM with 10% FBS before imaging. The imaging conditions were the same as in (a). (c) Number of cell division events of HeLa cells treated with RhoB-TCO and DiI-N_3 then Yale595-Tz and SiR-DBCO. Cells were washed and incubated in DMEM with 10% FBS before imaging. The imaging conditions were the same as in (a).

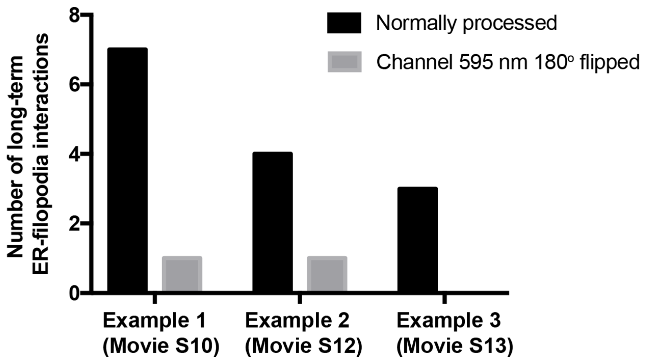

Supplementary Figure 18. Quantification of long-term ER-filopodia interactions. Black columns indicate the number of long-term ER-filopodia interactions that are preserved through out each movie. Gray columns indicate when the 595 nm channel was 180° flipped, the number of long-term ER-filopodia interacrtions that are preserved through out each movie.

NMR Spectrum:

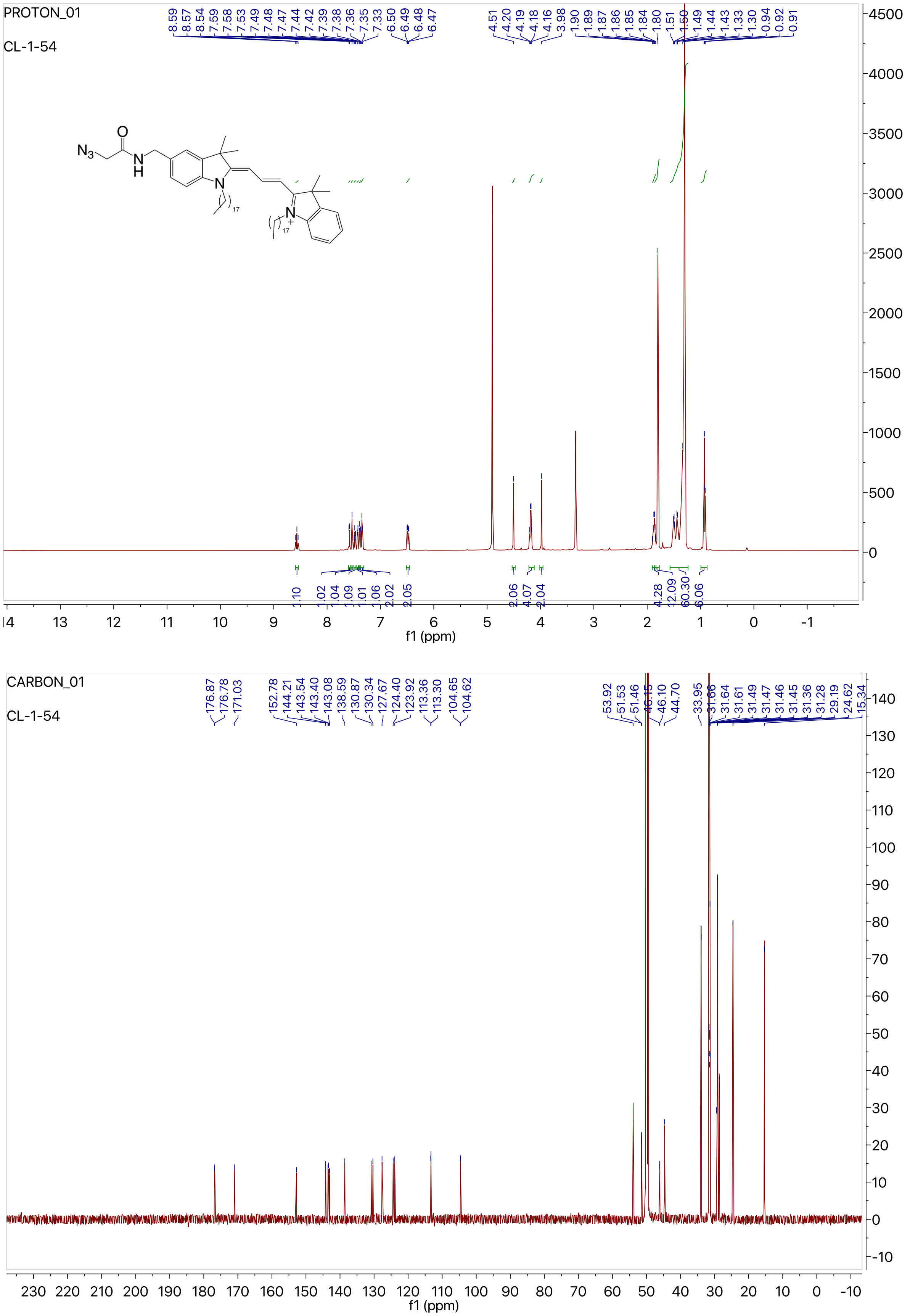

Supplementary Figure 19. **^{1}H- and ^{13}C-NMR spectrums of DiI-N_3.**

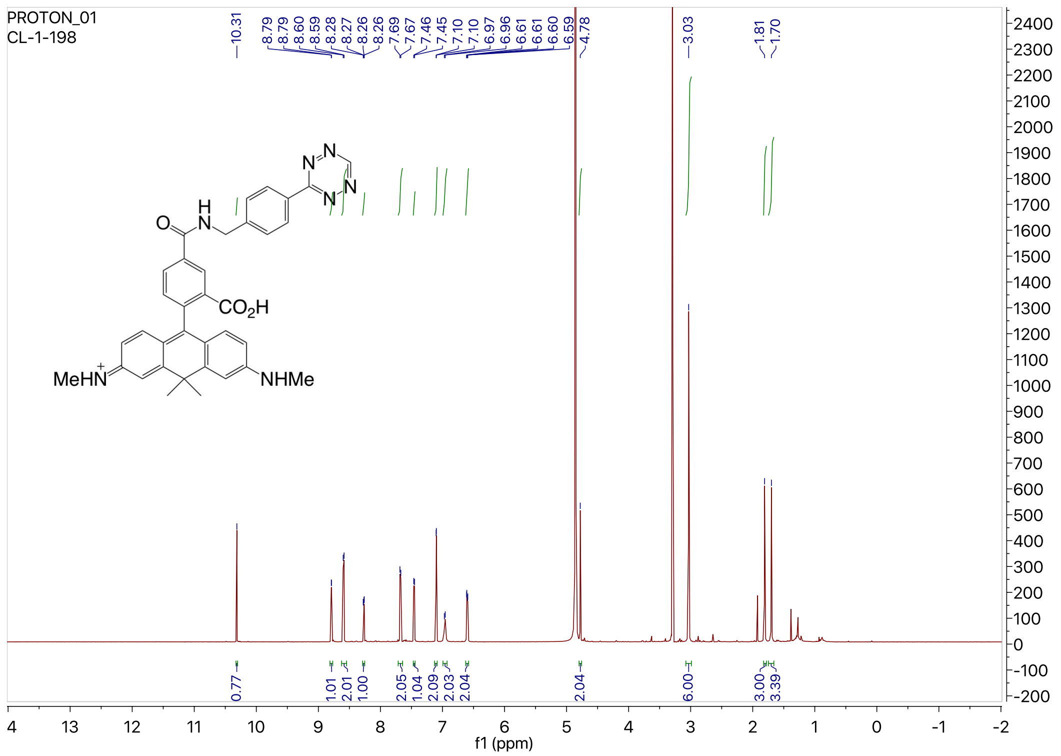

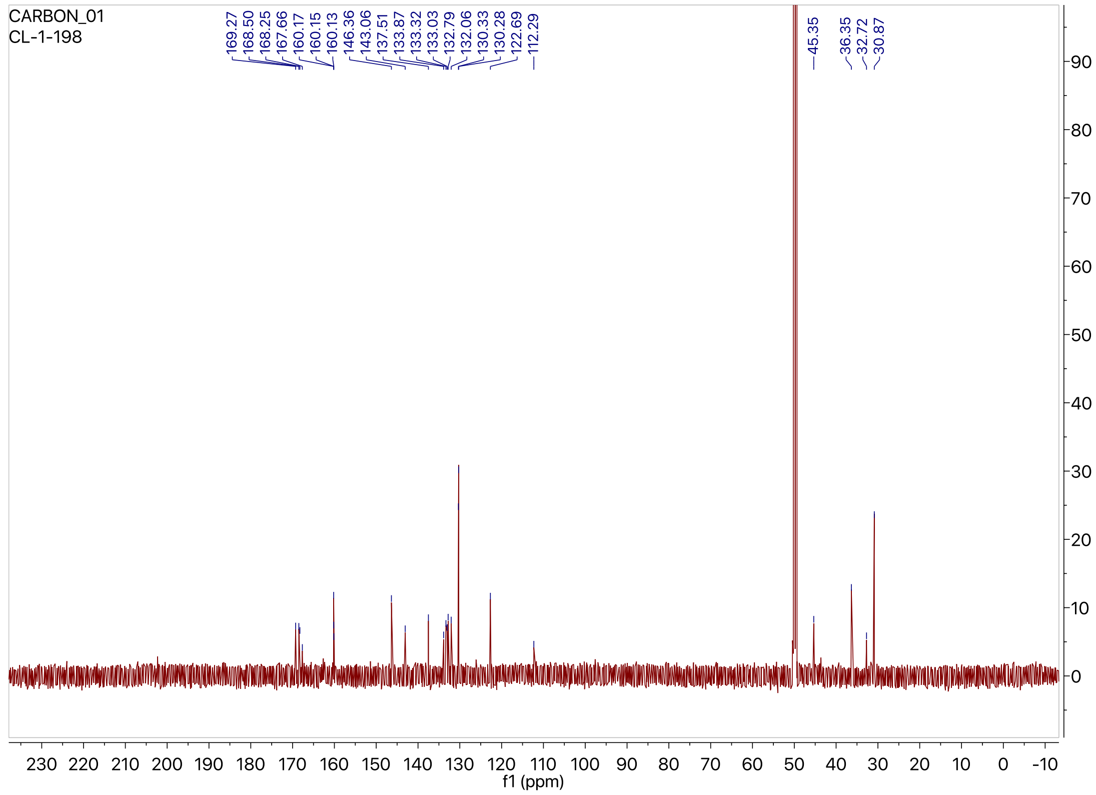

Supplementary Figure 20. ^{1}H- and ^{13}C-NMR spectrums of 580CP-Tz.

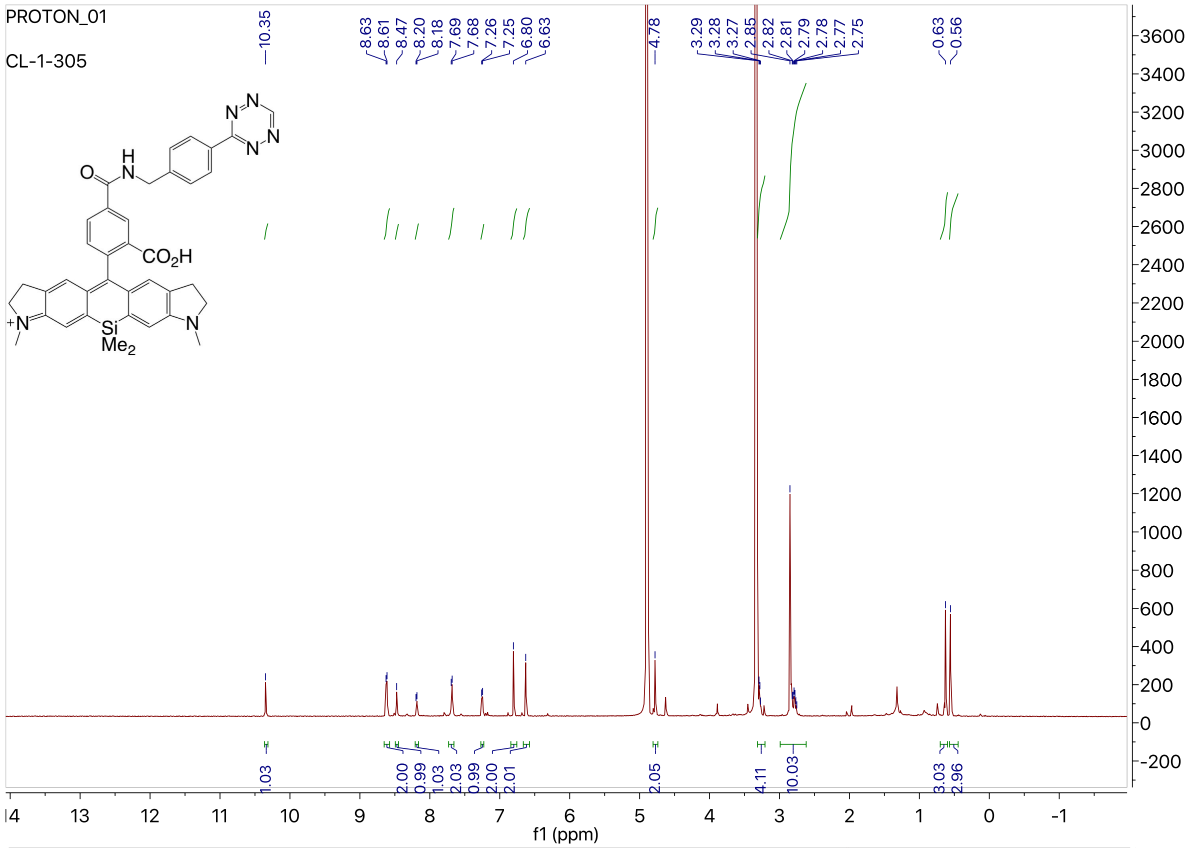

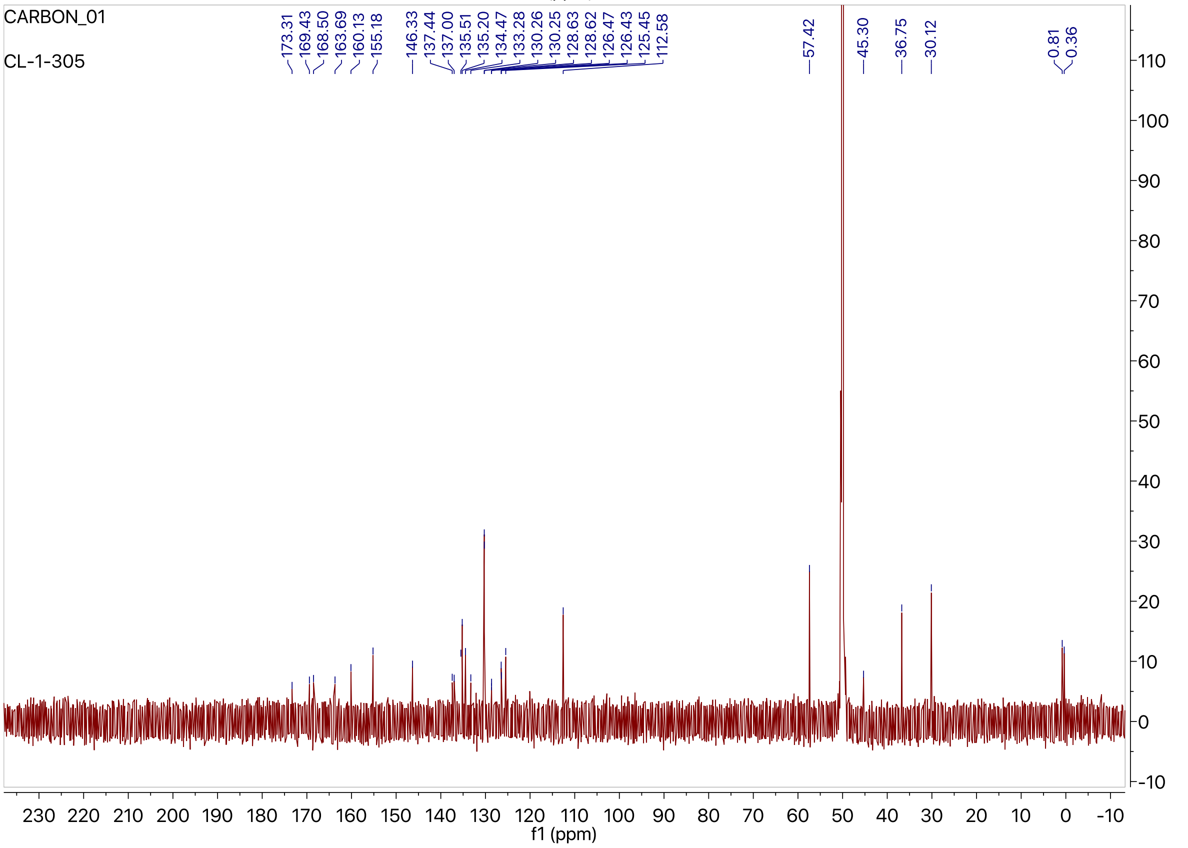

Supplementary Figure 21. ^{1}H- and ^{13}C-NMR spectrums of SiR700-Tz.

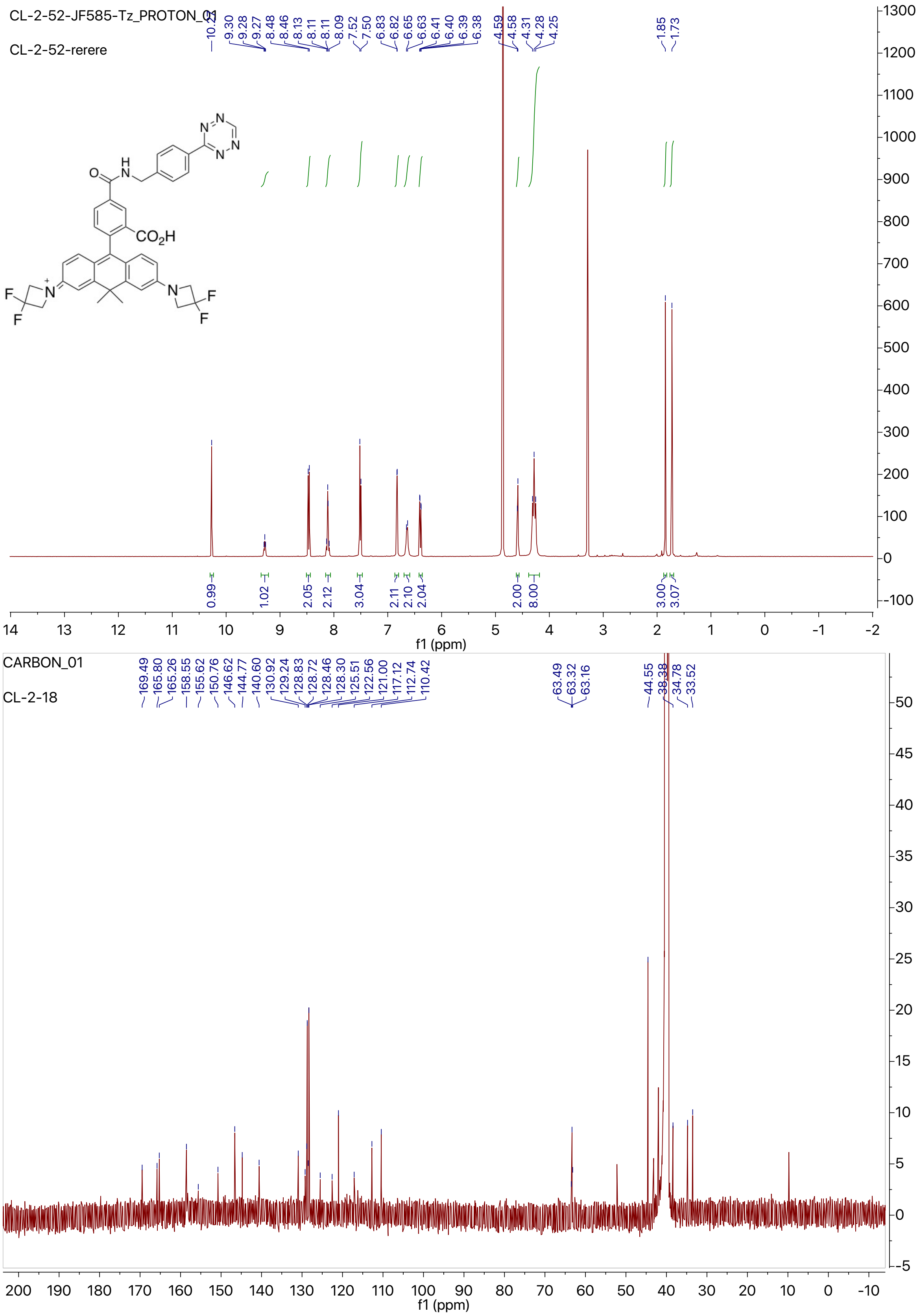

Supplementary Figure 22. ¹H- and ¹³C-NMR spectrums of JF585-Tz.

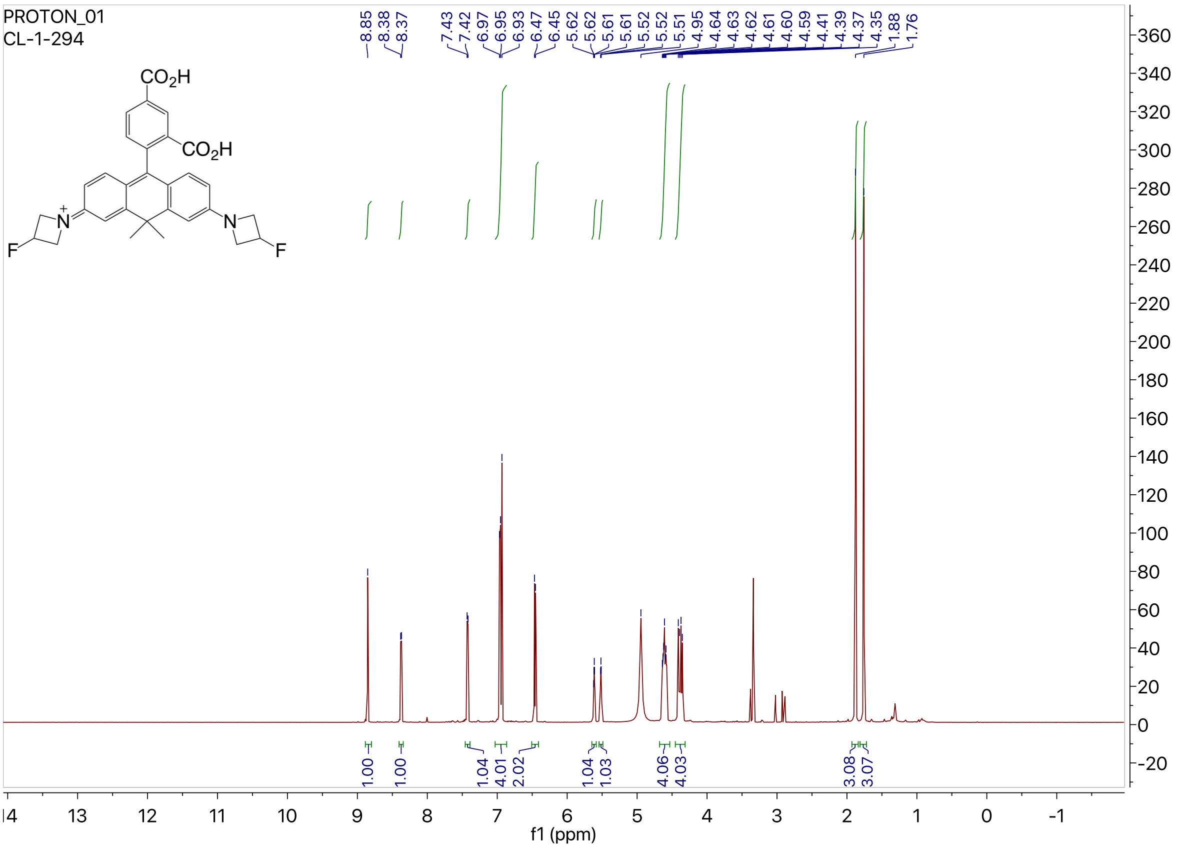

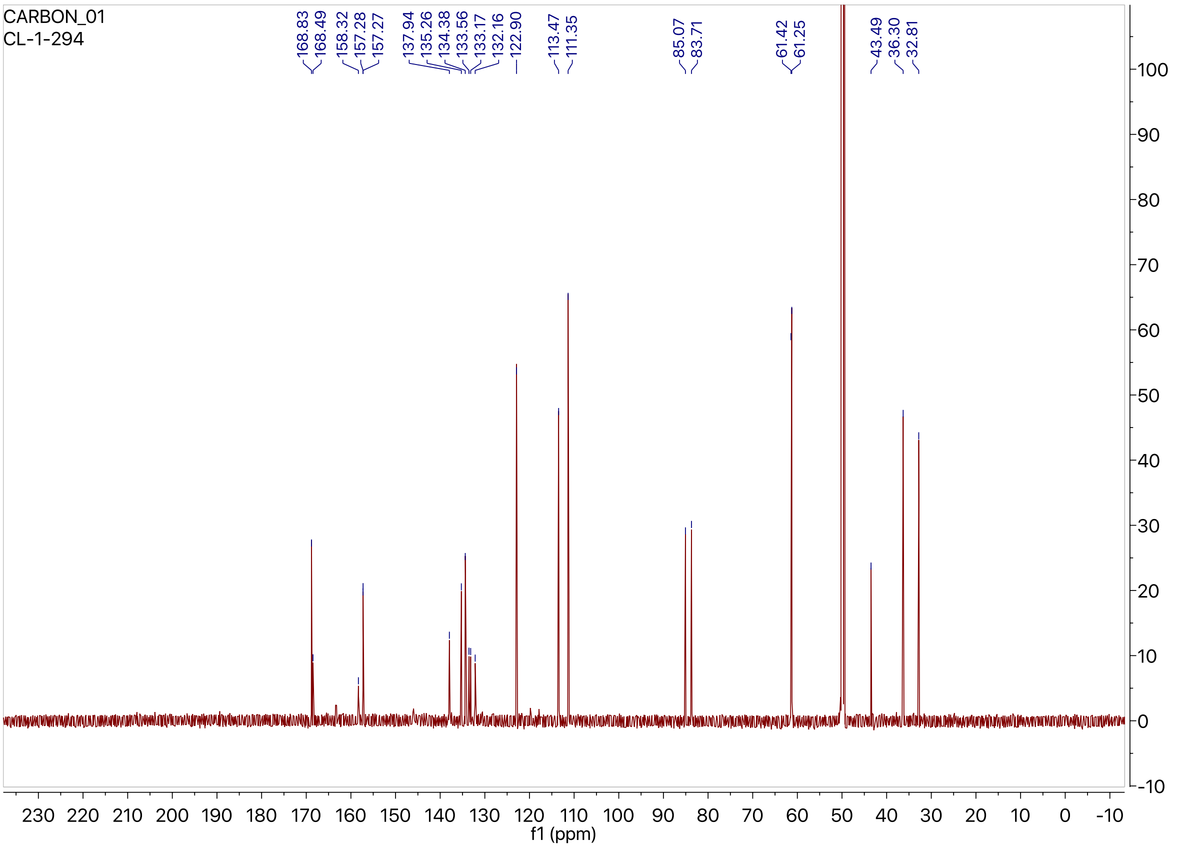

Supplementary Figure 23. ^{1}**H- and** ^{13}**C-NMR spectrums of Yale595-COOH.**

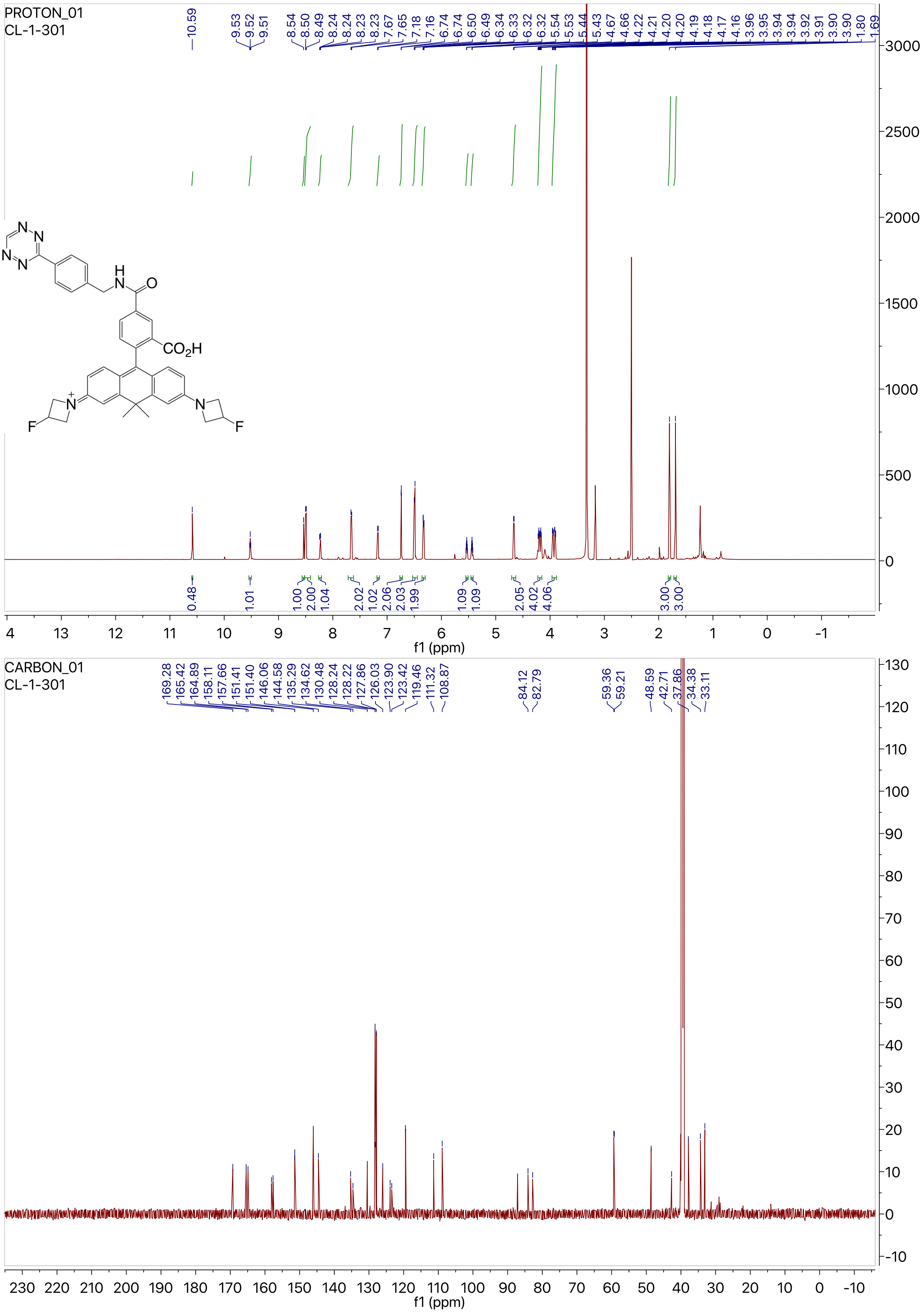

Supplementary Figure 24. ^{1}**H- and** ^{13}**C-NMR spectrums of Yale595-Tz.**

Supplementary References:

1. Erdmann, R.S. et al. Super-Resolution Imaging of the Golgi in Live Cells with a Bioorthogonal Ceramide Probe. *Angew Chem Int Edit* **53**, 10242-10246 (2014).
2. Takakura, H. et al. Long time-lapse nanoscopy with spontaneously blinking membrane probes. *Nat Biotechnol* **35**, 773-780 (2017).
3. Bottanelli, F. et al. Two-colour live-cell nanoscale imaging of intracellular targets. *Nat Commun* **7** (2016).
4. Lukinavicius, G. et al. A near-infrared fluorophore for live-cell super-resolution microscopy of cellular proteins. *Nat Chem* **5**, 132-139 (2013).
5. Butkevich, A.N. et al. Fluorescent Rhodamines and Fluorogenic Carbopyronines for Super-Resolution STED Microscopy in Living Cells. *Angew Chem Int Edit* **55**, 3290-3294 (2016).
6. Lukinavicius, G. et al. Fluorogenic Probes for Multicolor Imaging in Living Cells. *J Am Chem Soc* **138**, 9365-9368 (2016).
7. Grimm, J.B. et al. A general method to fine-tune fluorophores for live-cell and in vivo imaging. *Nat Methods* **14**, 987-994 (2017).
