# Supplementary material for: Two-color nanoscopy of organelles for extended times with HIDE probes
Source: Nat Commun. 2020 Aug 26;11:4271. doi: 10.1038/s41467-020-17859-1 (PMC7450044; doi:10.1038/s41467-020-17859-1)
Supplement: Supplementary file 2 — Description of Additional Supplementary Files [file 41467_2020_17859_MOESM2_ESM.pdf]

Description of Additional Supplementary Files

File Name: Supplementary Movie 1
Description: Plasma membrane of HeLa cells labeled with DiI-N_3 and SiR-DBCO. The movie shows long time-lapse live-cell imaging of plasma membrane under an SP8 STED microscope. Imaging was acquired at 2 seconds per frame. Scale bar: 2 µm.

File Name: Supplementary Movie 2
Description: Plasma membrane of HeLa cells labeled with DiI-TCO and CP580-Tz. The movie shows long time-lapse live-cell imaging of plasma membrane under an SP8 STED microscope. CP580-Tz photobleached quickly under the imaging conditions. Imaging was acquired at 2 seconds per frame. Scale bar: 2 µm.

File Name: Supplementary Movie 3
Description: Plasma membrane of HeLa cells labeled with DiI-TCO and SiR700-Tz. The movie shows long time-lapse live-cell imaging of plasma membrane under an SP8 STED microscope. SiR700-Tz photobleached quickly under the imaging conditions. Imaging was acquired at 2 seconds per frame. Scale bar: 2 µm.

File Name: Supplementary Movie 4
Description: Plasma membrane of HeLa cells labeled with DiI-TCO and Atto590-Tz. The movie shows long time-lapse live-cell imaging of plasma membrane under an SP8 STED microscope. Atto590-Tz showed severe cytotoxicity, as indicated by the cell bubbling under the imaging conditions. Imaging was acquired at 2 seconds per frame. Scale bar: 2 µm.

File Name: Supplementary Movie 5
Description: HeLa cells stably expressing Smo-Halo labeled with SiR-CA. The movie shows long time-lapse live-cell imaging of plasma membrane under an SP8 STED microscope. SiR-CA photobleached quickly under the imaging conditions. Imaging was acquired at 2 seconds per frame. Scale bar: 2 µm.

File Name: Supplementary Movie 6
Description: Plasma membrane of HeLa cells labeled with DiI-TCO and Yale595-Tz. The movie shows long time-lapse live-cell imaging of plasma membrane under an SP8 STED microscope. Minimal photobleaching was observed under the imaging conditions. Imaging was acquired at 2 seconds per frame. Scale bar: 2 µm.

File Name: Supplementary Movie 7
Description: PM and ER of HeLa cells labeled with DiI-N_3 + Cer-TCO and Yale595-Tz + SiR-DBCO. The movie shows long time-lapse live-cell imaging of plasma membrane (Red) and endoplasmic reticulum (Green) under an SP8 STED microscope. Minimal photobleaching was observed under the imaging conditions. Imaging was acquired at 2 seconds per frame. Scale bar: 2 µm.

File Name: Supplementary Movie 8
Description: HeLa cells expressing Smo-Halo and Sec61b-SNAP labeled with Yale595-BG and SiR-CA. The movie shows long time-lapse live-cell imaging of plasma membrane (Red) and endoplasmic reticulum (Green) under an SP8 STED microscope. Both Yale595-BG and SiR-CA photobleached quickly under the imaging conditions. Imaging was acquired at 2 seconds per frame. Scale bar: 2 µm.

File Name: Supplementary Movie 9
Description: PM and mitochondria of HeLa cells labeled with DiI-N_3 + RhoB-TCO and Yale595-Tz + SiR-DBCO. The movie shows long time-lapse live-cell imaging of plasma membrane (Red) and mitochondria (Green) under an SP8 STED microscope. Minimal photobleaching was observed under the imaging conditions. Imaging was acquired at 2 seconds per frame. Scale bar: 2 µm.

File Name: Supplementary Movie 10
Description: PM and ER of HUVEC cells labeled with DiI-N_3 + Cer-TCO and Yale595-Tz + SiR-DBCO. The movie shows long time-lapse live-cell imaging of plasma membrane (Red) and endoplasmic reticulum (Green) under an SP8 STED microscope. Minimal photobleaching was observed under the imaging conditions. We observed persist interactions between the filipodia of one cell and the endoplasmic reticulum of another cell. Imaging was acquired at 2 seconds per frame. Scale bar: 2 µm.

File Name: Supplementary Movie 11
Description: PM and mitochondria of hippocampal neurons labeled with DiI-N_3 + RhoB-TCO and Yale595-Tz + SiR-DBCO. The movie shows long time-lapse live-cell imaging of plasma membrane (Red) and mitochondria (Green) under an SP8 STED microscope. Minimal photobleaching was observed under the imaging conditions. Imaging was acquired at 2 seconds per frame. Scale bar: 0.5 µm.

File Name: Supplementary Movie 12
Description: Additional example 1 showing long-term intercellular interaction of filopodia and ER in HUVEC. The movie shows long time-lapse live-cell imaging of plasma membrane (Red) and endoplasmic reticulum (Green) under an SP8 STED microscope. Minimal photobleaching was observed under the imaging conditions. We observed persist interactions between the filipodia of one cell and the endoplasmic reticulum of another cell. Imaging was acquired at 2 seconds per frame. Scale bar: 1 µm.

File Name: Supplementary Movie 13
Description: Additional example 2 showing long-term intercellular interaction of filopodia and ER in HUVEC. The movie shows long time-lapse live-cell imaging of plasma membrane (Red) and endoplasmic reticulum (Green) under an SP8 STED microscope. Minimal photobleaching was observed under the imaging conditions. We observed persist interactions between the filipodia of one cell and the endoplasmic reticulum of another cell. Imaging was acquired at 2 seconds per frame. Scale bar: 1 µm.
